# A New Megastigmane Sesquiterpenoid from *Zanthoxylum Schinifolium* Sieb. et Zucc

**DOI:** 10.3390/molecules21030383

**Published:** 2016-03-19

**Authors:** Linzhen Hu, Kongchao Wang, Zhenzhen Wang, Junjun Liu, Kaiping Wang, Jinwen Zhang, Zengwei Luo, Yongbo Xue, Yu Zhang, Yonghui Zhang

**Affiliations:** 1 Union Hospital Affiliated to Tongji Medical College, Huazhong University of Science and Technology, Wuhan 430022, Hubei, China; hlz198@126.com; 2 Hubei Key Laboratory of Natural Medicinal Chemistry and Resource Evaluation, School of Pharmacy, Tongji Medical College, Huazhong University of Science and Technology, Wuhan 430030, Hubei, China; cs18379135010@163.com (K.C.W.); wzz75283@163.com (Z.Z.W.); junjun.liu@hust.edu.cn (J.J.L.); wkpzcq@163.com (K.P.W.); luozengwei@hust.edu.cn (Z.W.L.); yongboxue@hust.edu.cn (Y.G.X.); 3 Tongji Hospital Affiliated to Tongji Medical College, Huazhong University of Science and Technology, Wuhan 430030, Hubei, China; tjzhangjinwen@163.com

**Keywords:** *zanthoxylum schinifolium*, megastigmane, sesquiterpenoid, Kaposi’s sarcoma associated herpes virus

## Abstract

*Zanthoxylum schinifolium* Sieb. et Zucc. (Rutaceae), a dioecious shrub with hooked prickly branches, has been used as folk medicine for the treatment of the common cold, stomach ache, diarrhea, and jaundice in China, Korea, and Japan. In our phytochemical investigations on this genus, a new megastigmane sesquiterpenoid, which is referred to as schinifolenol A (**1**), was isolated from *Z. schinifolium*. The stereochemistry was characterized via the analyses of extensive spectra. The absolute configuration was established by the application of a modified Mosher’s experiment and assisted by a time-dependent density functional theory (TD-DFT) on calculated electronic circular dichroism (ECD). Bioactivity screenings showed that compound **1** exhibited a safe hypotoxicity and a better selectivity on anti-Kaposi’s sarcoma associated herpes virus (KSHV).

## 1. Introduction

**Figure 1:**
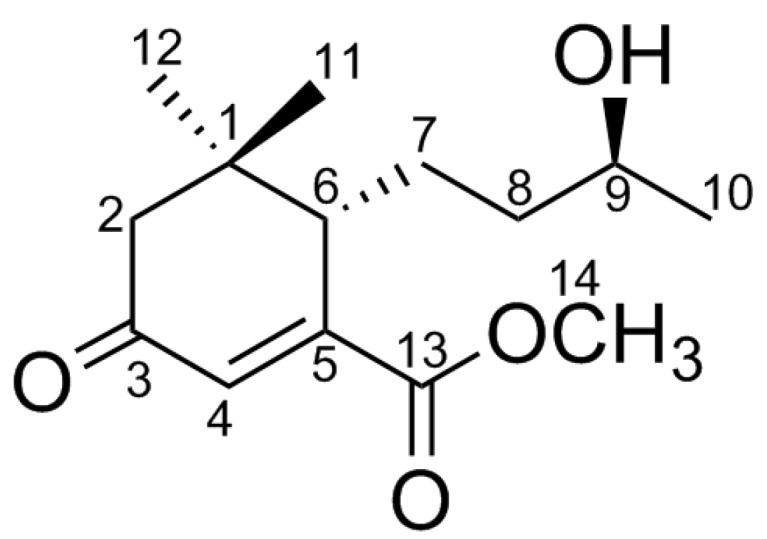
Structure of compound **1**.

*Zanthoxylum schinifolium* Sieb. et Zucc. is a dioecious shrub with hooked prickly branches from the genus *Zanthoxylum* (family Rutaceae), which was termed Qinghuajiao, Yajiao, Tianjiao, and Xiaohuajiao, *etc.*, and prosperously distributed from the south of the Yangtze River to the southwest provinces in China [1]. Clinical application in folk medicines of *Z. schinifolium* mainly included cures for the common cold, stomach ache, diarrhea, and jaundice in China, Korea, and Japan [2]. Recently, phytochemical studies on *Zanthoxylum* have resulted in the isolation of diverse chemical constituents such as alkaloids, amides, lignans, coumarins, essential oils and aliphatic acids [1,3,4,5]. In our continuous research for structurally unique and biologically active metabolites from traditional Chinese medicine [6,7,8], a megastigmane sesquiterpenoid termed schinifolenol A (**1**) (Figure 1) was obtained from the dried rhizomes of *Z.* *schinifolium*. Herein, we elucidated the isolation procedures and the stereochemistry structure establishment of compound **1**, as well as its inhibitory effect on Kaposi’s sarcoma associated herpes virus (KSHV) infection.

## 2. Results

The dried rhizomes of *Z. schinifolium* (30 kg) were exhaustively extracted with 95% EtOH to furnish a syrup (1.5 kg), which was successively partitioned by petroleum ether, CH_2_Cl_2_, and EtOAc against water. The petroleum ether fraction (300 g) was repeatedly subjected to silica gel column chromatography (silica gel CC), Sephadex LH-20, and semi-preparative High Performance Liquid Chromatography (HPLC) to afford a new megastigmane sesquiterpenoid, *viz*., compound **1**, which is named schinifolenol A (Figure 1).

Schinifolenol A (**1**), a violet oil, has the elemental composition of C_14_H_22_O_4_, which corresponded to the (+)-HRESIMS peak (*m/z* 255.1596 [M + H]^+^, calcd as 255.1592). IR (KBr) spectrum showed the characteristic absorption bands for hydroxyl (3695 cm^−1^) and carbonyl (1724 and 1667 cm^−1^) functionalities along with UV (CH_3_OH) spectrum at λ_max_ 244 nm, which are closely similar with those data of megastigmane sesquiterpenoids such as blumenol C in reported literatures [9,10,11]. According to the NMR data of the literature [11], the difference between compound **1** and blumenol C is that a methoxycarbonyl group at C-5 of **1** was replaced by a methyl function in blumenol C. Spectral analyses of 1D NMR (Table 1) and HSQC correlations indicated the presence of two carbonyls (δ_C_ 202.3 and 169.3), one oxygenated methyl (δ_H_ 3.84, s and δ_C_ 53.4), three methyls (δ_H_ 1.01, s; 1.12, d, *J* = 6.2 Hz; and 1.15, s), three methylenes, three methines (including an olefinic methine (δ_H_ 6.55, s and δ_C_ 131.4) and a hydroxylated carbon (δ_H_ 3.65, m and δ_C_ 68.8)), and two quaternary carbons (including one olefinic carbon (δ_C_ 155.2)). Referring to the literature data [9,10,11], the aforementioned analyses illustrate that compound **1** belongs to a class chemicals of megastigmane sesquiterpenoid.

**Figure 2:**
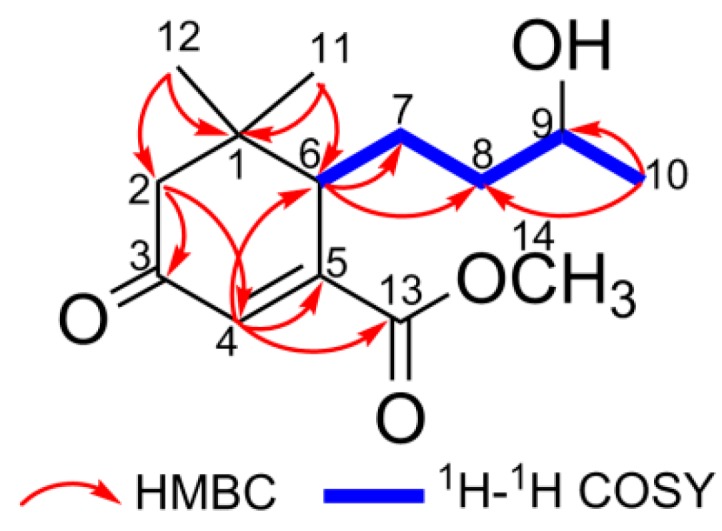
Key HMBC and ^1^H-^1^H COSY correlations of compound **1**.

Combining with the signals of HSQC, iterative analyses of HMBC and ^1^H-^1^H COSY experiments could elucidate the planar constitution of **1**. HMBC correlations from Me-12 and Me-13 to C-1, C-2, and C-6, from H-2 to C-3 and C-4, and from H-4 to C-5 and C-6 indicated the presence of a 5,5-dimethyl-cyclohex-3-one entity. Meanwhile, the HMBC cross peak of H-4 to the ester carbonyl C-13 implied that the ester carbonyl function was located at C-5. Moreover, HMBC correlations from Me-10 to C-8 and C-9, and from H-6 to C-7 and C-8, as well as the ^1^H-^1^H COSY spin systems of H-6/H-7/H-8/H-9/H-10 revealed that a 3-hydroxybutyl functionality was connected to the 3,3-dimethyl-cyclohexanone at C-6 (Figure 2). No diagnostic NOESY could be applied to determine the relative configurations of C-6 and C-9, since both carbons were located on a rotational aliphatic chain. Thus, the aforementioned spectral analyses allowed us to assign compound **1** as a member of megastigmane sesquiterpenoid.

**Figure 3:**
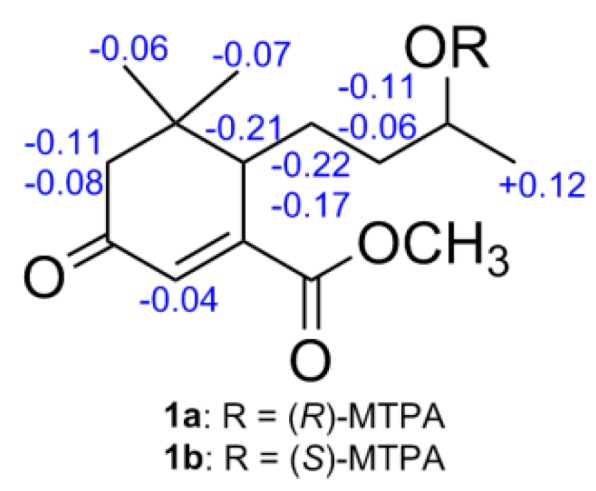
∆δ values (in ppm) = δ*_S_*_-MTPA-ester_ − δ*_R-_*_MTPA-ester_ for **1a** and **1b**.

**Figure 4:**
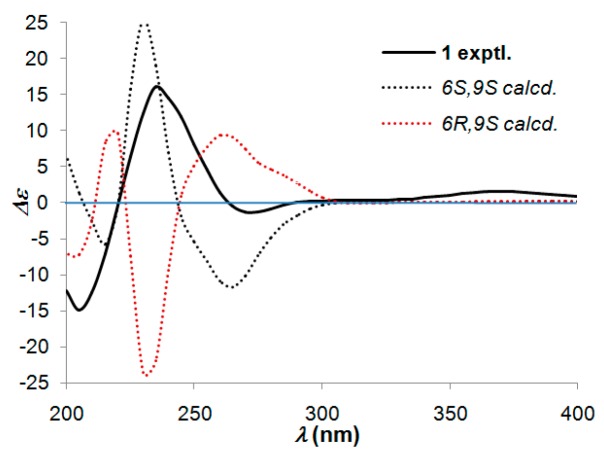
Experimental and calculated ECD spectra of **1**.

In order to determine the absolute configuration of compound **1**, a modified Mosher’s method was carried out to establish the stereochemistry characteristic of secondary alcohol carbon C-9. (*S*)- and (*R*)-MTPA esters of **1** were prepared as previously reported [12,13], then analyzed via ^1^H-NMR chemical shifts study. The distinguishable values (∆δ = δ_S-MTPA-ester_ − δ_R-MTPA-ester_) were calculated for proton chemical shifts adjacent to C-9, as shown in Figure 3. Correspondingly, the absolute configuration at C-9 was confirmed to be *S*. Furthermore, a time-dependent density functional theory (TD-DFT) method on ECD calculation was performed to determine the absolute configuration of the other chiral carbon, *viz*., C-6. Based on the ascertained absolute configuration of C-9, the calculated ECD curve of (6*S*,9*S*)-**1** showed a good consistency with the experimental ECD curve (Figure 4), which unequivocally established the absolute configuration of **1** as 6*S*,9*S*.

Since previous literatures reported that dictamnine (one type of alkaloids was isolated from *Z. schinifolium*) exhibited activity towards anti-Epstein–Barr virus (EBV) [14] and sesquiterpenes/sesquiiterpene lactones exhibited moderately activities towards anti-HIV [15], these two assays were also assessed for compound **1** and neither of the results showed obvious activities (**1** towards both assays with CC_50_ > 300 μM and EC_50_ > 300 μM). Besides the aforementioned assays of anti-EBV and anti-HIV, some other bioactive screenings for **1** were performed, which included inhibitory activities on β-site amyloid precursor protein cleaving enzyme 1 (BACE1) (**1** towards this assay with IC_50_ > 40 μM), inhibitory activities on NO production (**1** towards this assay with IC_50_ > 25 μM), and cytotoxic activities against five human cancer cell lines (HL-60, SMMC-7721, A-549, MCF-7, and SW480) (**1** towards all these cytotoxicity assays with IC_50_ > 40 μM). Disappointingly, compound **1** did not show any activities in the above assays.

Kaposi’s sarcoma associated herpes virus (KSHV) belongs to the gamma 2-herpesvirus subfamily, which is the etiological agent of all types of Kaposi’s sarcoma, like primary effusion lymphoma, multicentric Castleman’s disease, and posttransplant [16]. Currently, typical antiviral drugs, like acyclovir or ganciclovir, do not show a satisfactory potency to extinguish gamma herpes viruses in human bodies [17]. In this case, it is a challenge to discover more effective drugs or lead compounds against KSHV. Compound **1** was used to carry out an anti-KSHV assay referring to the procedure of previous literature [18]. The inhibitory activity of compound **1** against KSHV lytic replication was measured. The results indicated that **1** exhibited potency with a safe hypotoxicity and a definite selectivity (*i.e.*, EC_50_ of 501.3 μM and selectivity index of 1.99, respectively).

## 3. Materials and Methods 

### 3.1. General Experiments

TLC was carried out by silica gel 60 F254 (Shanghai Beinuo Biological Technology Co. Ltd., Shanghai, China). Sephadex LH-20 (GE Healthcare Bio-Sciences AB, Uppsala, Sweden), Silica gel (200–300 mesh; Shanghai Xibao Biological Technology Co. Ltd., Shanghai, China), and RP-18 (50 μm, Merck Co. Ltd., Darmstadt, Germany) were applied in column chromatography. Pseudomolecular ion peak was recorded by analyzing HRESIMS data through a Thermo Fisher LC-LTQ-Orbitrap XL spectrometer (Thermo Fisher Scientific Inc., Waltham, MA, USA). A Perkin-Elmer 341 polarimeter (Perkin Elmer Inc., Waltham, MA, USA) was performed to measure optical rotation. The UV and IR spectra were obtained by a Varian Cary 50 (Varian Medical Systems, Salt Lake City, UT, USA) and Bruker Vertex 70 instruments (Brucker Corporation, Karlsruhe, Germany), respectively. The NMR spectra were acquired using a Bruker AM-600/400 spectrometer (Brucker Corporation). The chemical shifts of ^1^H- and ^13^C-NMR were referenced to the solvent peaks for methanol-d_4_ at δ_H_ 3.31 and δ_C_ 49.2. HPLC procedures were carried out on a Dionex Ultimate 3000 (Thermo Fisher Scientific Inc.) applied with a UV detector and a semi-preparative column (5 μm, 10 × 250 mm, Welch Ultimate^®^ XB-C_18_).

### 3.2. Plant Material

The dried rhizomes of *Z. schinifolium* were collected in September 2013 at Da-Bie Mountain area of Hubei Province, China and authenticated by Changgong Zhang. A voucher specimen (ID 20131011) has been preserved in Herbarium of Material Medicine, School of Pharmacy, Tongji Medical College, Huazhong University of Science and Technology, Wuhan, China.

### 3.3. Extraction and Isolation

The air-dried rhizomes of *Z. schinifolium* (30 kg) were exhaustively extracted with 95% EtOH at 25 °C and to furnish a syrup (1.5 kg) after vacuum distillation. The syrup was sequentially partitioned by petroleum ether, CH_2_Cl_2_, and EtOAc against water. Based on the TLC analyses, the petroleum ether extracts (300 g) were chromatographed and silica gel CC eluted with petroleum ether-acetone (100:1–1:1) to afford seven fractions (Fr.1–Fr.10). Fr.6 was further subjected to silica gel CC by gradient elution with petroleum ether–acetone (50:1–1:1), which afforded seven subfractions (Fr.6.1–Fr.6.7). Then, Fr.6.5 was subjected to MPLC (RP-18, CH_3_OH–H_2_O, 30%–60%) to yield three subfractions of Fr.6.5.1–Fr.6.5.3. Next, Fr.6.5.2 was subjected to Sephadex LH-20, repurified by a silica gel CC eluting with CH_2_Cl_2_–CH_3_OH 25/1, and isolated via semi-preparative HPLC (CH_3_OH-H_2_O 30%) to obtain **1** (9.2 mg).

### 3.4. Experimental Procedures of the Derivatives of (S)-MTPA and (R)-MTPA Esters of **1**

MTPA ester derivatives of **1** were obtained referring to the previously reported procedure [12,13]. A solvent of **1** (1.0 mg) in anhydrous CH_2_Cl_2_ (2.5 mL) was successively added with (*R*)-MTPA (25.0 mg), dimethylaminopyridine (17 mg), and trimethylamine (25 µL). Then, the mixed solution was agitated and refluxed at 25 °C until 2 h and extinguished whereby adding with 40 µL anhydrous CH_3_OH. Next, the reaction mixture underwent a vacuum evaporation to yield a residue, which was purified via a small silica gel CC (1.6 g, hexane-IPA (75:1–40:1), *v*/*v*)) to provide the (*S*)-MTPA ester of **1** (**1a**, 1.5 mg). The (*R*)-MTPA derivative (**1b**, 1.6 mg) was obtained using (*S*)-MTPA chloride and experienced via the same procedure.

Schinifolenol A (**1**): violet oil; ${\left[\alpha \right]}_{20}^{D}$ + 64.8 (*c* 0.03, CH_3_OH); UV (CH_3_OH) λ_max_ (log ε) 244 (3.84) nm; IR (KBr) ν_max_ 3695, 2965, 2361, 2337, 1724, 1667 cm^−1^; ECD (*c* 1.09 × 10^−3^ M, CH_3_OH) λ_max_ nm (∆ε) 235 (+16.04), 270 (−1.35); ^1^H- and ^13^C-NMR data, see Table 1; positive HRESIMS: *m*/*z* 255.1596 [M + H]^+^ (calcd for C_14_H_23_O_4_, 255.1592).

Compound **1a**: (*S*)-MTPA-ester: Amorphous powder; ^1^H-NMR (400 MHz, in methanol-*d*_4_) δ_H_: 7.49–7.51 (2H, m, aromatic protons), 7.39–7.41 (3H, m, aromatic protons), 6.53 (1H, s, H-4), 2.47 (1H, m, H-2a), 2.30 (1H, d, *J* = 17.5 Hz, H-6), 1.98 (1H, d, *J* = 17.6 Hz, H-2b), 1.68 (1H, m, H-7a), 1.63 (1H, m, H-8a), 1.56 (1H, m, H-8b), 1.34, (3H, d, *J* = 6.3 Hz, Me-10), 1.18 (1H, m, H-7b), 0.94 (3H, s, Me-11), 0.93 (3H, s, Me-12); positive HRESIMS: *m*/*z* 493.1806 [M + Na]^+^ (calcd for C_24_H_29_F_3_O_6_Na, 493.1814).

Compound **1b**: (*R*)-MTPA-ester: Amorphous powder; ^1^H-NMR (400 MHz, in methanol-*d*_4_) δ_H_: 7.48–7.50 (2H, m, aromatic protons), 7.41–7.45 (3H, m, aromatic protons), 6.57 (1H, s, H-4), 2.58 (1H, m, H-2a), 2.51 (1H, d, *J* = 17.5 Hz, H-6), 2.07 (1H, d, *J* = 17.6 Hz, H-2b), 1.89 (1H, m, H-7a), 1.74 (1H, m, H-8a), 1.62 (1H, m, H-8b), 1.35 (1H, m, H-7b), 1.25 (3H, d, *J* = 6.3 Hz, Me-10), 1.07 (3H, s, Me-11), 0.99 (3H, s, Me-12); positive HRESIMS: *m*/*z* 493.1802 [M + Na]^+^ (calcd for C_24_H_29_F_3_O_6_Na, 493.1814).

### 3.5. Anti-KSHV Assay

Anti-KSHV assay was evaluated via cytotoxicity assessments and anti-KSHV infectivity assays. Human iSLK.219 cells were adopted to assess the bioactivity of compound **1** towards anti-KSHV. The human iSLK.219 cells were embedded with the rKSHV.219 virus, which was a harbored green fluorescent protein (GFP), through control of the elongation factor 1α (EF-1α) promoter. The lytic replication of KSHV was activated by the addition with 1.2 mM sodium butyrate (NaB) (Sigma, Shanghai, China) and 1 µg/mL doxycycline (Dox) (Beyotime, Jiangsu, China) [19,20]. After cells grew to 70% confluence in 96-well culture plates, compound **1** in presence of Dox and NaB with assigned concentrations was instilled to the wells. According to AlamarBlue^®^ Cell Viability Assay (Invitrogen, Shanghai, China), the cell viability was evaluated after 48 h post drugs-induced. The luminescent expression was measured by the Envison 2102 Multilabel Reader (Perkin Elmer). The 50% cytotoxic concentration (CC_50_) of compound **1** was obtained through mathematical statistics with Graphpad5.0 Prism. The result was shown in Figure S1, Supplementary Materials.

An infectivity assay, like previous reports [18], was performed to determine the anti-KSHV activity of compound **1**. The supernatants were harvested from iSLK.219-treated or untreated with compound **1** containing Dox and NaB at 48 h. Then, the supernatants were added to infect the Vero cells, which were seeded in a 96-well plate. Next, the Vero cells were centrifuged by a SORVALL^®^Pico apparatus at 1500× *g* for 60 min [21]. The supernatants were replaced by fresh DMEM medium to remove superfluous viruses. At 48 h, the expression of GFP per well in Vero cells were detected via an Operetta High-Content Screening System (HCS) (Perkin Elmer). Image fields (9/well) were observed by the automated microscope based HCS. The GFP intensity of each well was afforded using the Harmony 3.5 software (Perkin Elmer). The DMSO control was used as normalized group. The 50% effective concentration (EC_50_) of compound **1** towards anti-KSHV infectivity was calculated by reduced quantitative expression of the intensity of GFP by 50%. The result is exhibited in Figure S1, Supplementary Materials.

## 4. Conclusions

A new megastigmane sesquiterpenoid, methyl (*S*)-6-((*S*)-3-hydroxybutyl)-5,5-dimethyl-3-oxocyclohex-1-ene-1-carboxylate (**1**), termed schinifolenol A, was discovered from the rhizomes of *Zanthoxylum schinifolium*. The absolute configuration was established by the analyses of the extensive spectra including HRESIMS, NMR, UV, and IR spectra, the application of the modified Mosher’s method, and the method of calculated ECD spectra using the time-dependent density functional theory. Bioactivity screenings suggested that compound **1** had potential activity on anti-KSHV infection with a safe hypotoxicity and a definite selectivity.

## Figures and Tables

**Table 1:** ^1^H-NMR (400 MHz) and ^13^C-NMR (100 MHz) Spectral Data of Compound **1** in Methanol-*d*_4_ (δ in ppm, *J* in Hz).

NO.	δ_H_	δ_C_	NO.	δ_H_	δ_C_
1		37.1	8	1.50 m, 1.37 m	39.1
2	2.63d (6.4), 2.09d (17.6)	48.1	9	3.65 m	68.8
3		202.3	10	1.12d (6.2)	23.5
4	6.55 s	131.4	11	1.15 s	28.0
5		155.2	12	1.01 s	28.7
6	2.60d (4.8)	46.4	13		169.3
7	2.00 m, 1.36 m	28.8	14	3.84 s	53.4

## Supplementary Materials

Supplementary materials can be accessed at: http://www.mdpi.com/1420-3049/21/3/383/s1.

## Abbreviations

The following abbreviations are used in this manuscript:

*Z.**schinifolium*: *Zanthoxylum schinifolium*
TD-DFT: time-dependent density functional theory
ECD: electronic circular dichroism
KSHV: Kaposi’s sarcoma associated herpesvirus
HRESIMS: High-resolution electrospray ionization mass spectra
CC: column chromatography
HPLC: High Performance Liquid Chromatography
EtOH: ethanol
EtOAc: ethyl acetate
MTPA: α-Methoxy-α-Trifluoromethylphenylacetic acid
IPA: isopropanol
