# Supplementary material for: A New Megastigmane Sesquiterpenoid from Zanthoxylum Schinifolium Sieb. et Zucc
Source: Molecules. 2016 Mar 19;21(3):383. doi: 10.3390/molecules21030383 (PMC6273384; doi:10.3390/molecules21030383)
Supplement: Supplementary file 1 [file molecules-21-00383-s001.pdf]

SupplementaryMaterials: A New Megastigmane Sesquiterpenoid from *Zanthoxylum schinifolium* Sieb. et Zucc.

Linzhen Hu, Kongchao Wang, Zhenzhen Wang, Junjun Liu, Kaiping Wang, Jinwen Zhang, Zengwei Luo, Yongbo Xue, Yu Zhangand Yonghui Zhang

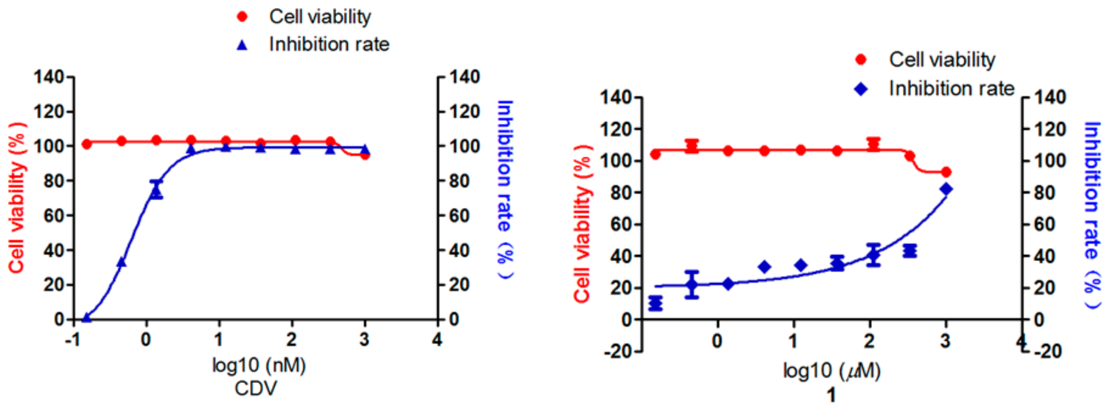

Figure S1. The effects of positive control cidofovir (CDV) and 1 on human iSLK.219 cells viabilities and the inhibitory effects of CDVand 1 on lytic replication of KSHV infecting Vero cells were measured *in vitro*.

Computational details

Conformational analyses were performed through both BALLOON and confab programs [1,2] in order to establish the absolute structures of compound **1**.The BALLOON program explores conformational spaces with genetic algorithm, and synchronously, the confab program systematically generates diverse low energy conformations that are proposed to be close to crystal structures. The conformations generated by the above programs were assembled together by the removal of duplicated conformations whose root mean square (RMS) distance was less than 0.5 Å. Semi-empirical PM3 quantum mechanical geometry optimizations were executed on conformations through the Gaussian 09 program [3]. Duplicated conformations after geometry optimization were subsequently identified and disposed. Remaining conformations were further optimized at B3LYP/6-31G* level of theory in methanol solvent with IEFPCM3 solvation model using Gaussian 09 program [4], and duplicated conformations presenting after these calculations were removed according to the same RMS criteria above. Harmonic vibrational frequencies were fulfilled to build the stability of the finally obtained conformers. Oscillator strengths and rotational strengths of 20 weakest electronic excitations of each conformer were calculated by the TDDFT methodology at the B3LYP/6-311++G** level of theory adopting methanol as solvent by the IEFPCM solvation model carried out in Gaussian 09 program. The ECD spectra data for each conformer were then simulated by using a Gaussian function with a band width σ of 0.45 eV. Calculated spectra for each conformation were combined after Boltzmann weighting according to their population contribution.

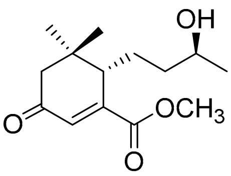

1A

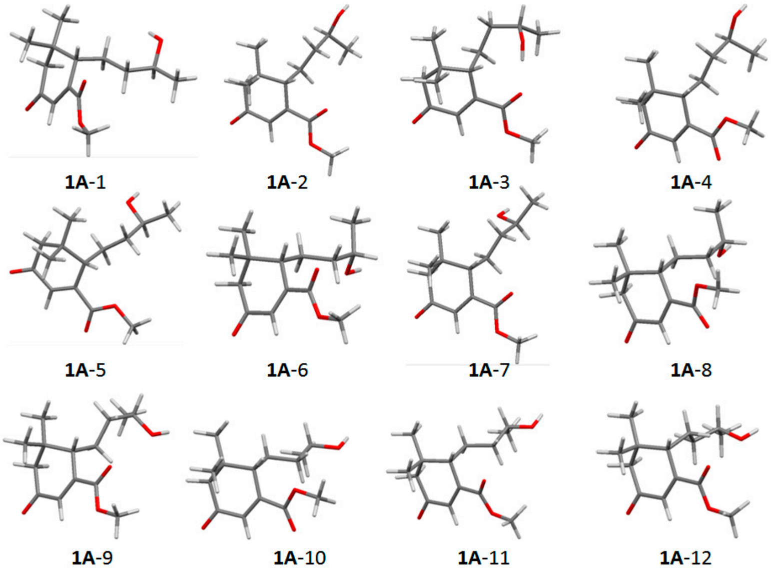

Figure S2. Optimized geometries of predominant conformers for compound **1A** at the B3LYP/ 6-31G(d,p) level in methanolsolution.

Table S1. Important thermodynamic parameters (a.u.) and Boltzmann distributions of the optimized compound **1A**at B3LYP/6-31G(d,p) level in methanol solution.

Conformations	E+ZPE	G	%
**1A**-1	−847.295352	−847.344421	26.97
**1A**-2	−847.293642	−847.342902	5.40
**1A**-3	−847.295790	−847.342584	3.86
**1A**-4	−847.293279	−847.342099	2.31
**1A**-5	−847.294839	−847.343725	12.91
**1A**-6	−847.293883	−847.342450	3.35
**1A**-7	−847.295339	−847.344153	20.31
**1A**-8	−847.293124	−847.341831	1.74
**1A**-9	−847.293036	−847.342160	2.46
**1A**-10	−847.294040	−847.342704	4.38
**1A**-11	−847.294787	−847.343628	11.65
**1A**-12	−847.294309	−847.342766	4.68

E+ZPE, G: total energy with zero point energy (ZPE) and Gibbs free energy in methanol solutionat B3LYP/6-31G(d,p) level. %: Boltzmann distributions, using the relative Gibbs free energies as weighting factors.

Table S2. Optimized Z-matrixes of compound **1A** in methanol solution (Å) at B3LYP/6-31G(d,p) level.

**1A-1**				**1A-2**			
C	1.64605	1.041489	−0.907893	C	2.27048	0.064353	−0.717907
C	0.720393	0.821755	0.047919	C	1.230992	0.481331	0.032213
C	2.550444	−0.025196	−1.384676	C	2.440002	−1.35297	−1.09861
C	−0.060066	1.972224	0.612263	C	1.094054	1.924472	0.42233
C	2.395934	−1.401726	−0.762446	C	1.344359	−2.314258	−0.67872
C	0.463994	−0.546448	0.650154	C	0.115724	−0.431994	0.503419
C	1.776853	−1.399791	0.655765	C	0.617585	−1.908962	0.625671
C	1.508058	−2.852845	1.08976	C	−0.538035	−2.894058	0.877804
C	2.775697	−0.788679	1.664259	C	1.593274	−2.017626	1.819566
C	−4.490143	−0.37441	−0.745553	C	−3.773945	1.243972	−1.361445
C	−0.830389	4.178311	0.268231	C	2.011247	4.096251	0.294187
C	−0.731244	−1.238273	−0.068227	C	−1.129767	−0.213913	−0.42058
C	−2.038215	−0.432946	−0.099886	C	−2.457894	−0.147475	0.345685
C	−3.212926	−1.206624	−0.721027	C	−3.69522	−0.050969	−0.551478
O	3.384005	0.20561	−2.255538	O	3.417655	−1.714517	−1.746615
O	−0.551451	1.967034	1.727326	O	0.176138	2.349438	1.103029
O	−3.516831	−2.397671	0.023204	O	−4.822405	−0.164345	0.331423
O	−0.143352	3.012767	−0.232621	O	2.084057	2.698522	−0.050805
H	1.800902	2.027972	−1.332832	H	3.038368	0.751254	−1.057405
H	1.764408	−1.986451	−1.447336	H	0.625955	−2.354784	−1.511208
H	3.377936	−1.887846	−0.765497	H	1.778033	−3.316752	−0.596343
H	0.161092	−0.388724	1.692117	H	−0.169311	−0.092572	1.505943
H	2.456683	−3.391508	1.197867	H	−1.068304	−2.652838	1.806224
H	0.900754	−3.401317	0.363227	H	−0.142477	−3.911774	0.977442
H	0.994974	−2.884816	2.058718	H	−1.265313	−2.903198	0.059912
H	3.713125	−1.35672	1.662861	H	2.453864	−1.348425	1.720443
H	2.364577	−0.823839	2.679938	H	1.97421	−3.041647	1.908587
H	3.01745	0.254768	1.438392	H	1.082281	−1.768091	2.756683
H	−4.342244	0.546471	−1.319343	H	−2.945973	1.326143	−2.073911
H	−5.308078	−0.941144	−1.201964	H	−4.706654	1.281265	−1.937895
H	−4.786988	−0.10207	0.274168	H	−3.748032	2.113836	−0.694607
H	−0.806897	4.899987	−0.547234	H	2.882633	4.554859	−0.171467
H	−0.316065	4.573988	1.147103	H	2.04422	4.221481	1.378959
H	−1.860492	3.926253	0.529981	H	1.090251	4.535624	−0.096046
H	−0.445237	−1.492563	−1.097989	H	−1.001829	0.721246	−0.975677
H	−0.922828	−2.181199	0.453818	H	−1.168555	−1.003888	−1.181257
H	−2.31811	−0.132688	0.91819	H	−2.579161	−1.03498	0.976203
H	−1.902382	0.488366	−0.681632	H	−2.441329	0.719778	1.019221
H	−2.947281	−1.482287	−1.754441	H	−3.690531	−0.906507	−1.247862
H	−2.820901	−3.047593	−0.158942	H	−5.623063	−0.111742	−0.215019
**1A-3**				**1A-4**			
C	1.774041	1.317046	−0.459734	C	2.316713	0.626231	−0.816317
C	0.582183	0.875867	−0.008607	C	1.188449	0.842399	−0.111643
C	2.901958	0.40019	−0.724377	C	2.857948	−0.727927	−1.04032
C	−0.516812	1.856326	0.273343	C	0.761145	2.27554	0.053433
C	2.641846	−1.080627	−0.537554	C	2.039316	−1.889069	−0.509114
C	0.255001	−0.591406	0.205755	C	0.335224	−0.271916	0.467191
C	1.55822	−1.403675	0.518357	C	1.201854	−1.547083	0.74583
C	1.317883	−2.924017	0.518692	C	0.335895	−2.765951	1.111515
C	2.069709	−1.014243	1.924479	C	2.143218	−1.268821	1.939703

C	−3.774529	−0.682557	−1.121195	C	−3.818855	0.126884	−1.53235
C	−1.192177	4.120489	0.288607	C	−0.858374	3.729087	0.973221
C	−0.577312	−1.098736	−1.02336	C	−0.903908	−0.486763	−0.466632
C	−1.679923	−2.135415	−0.718168	C	−2.218931	−0.700635	0.295004
C	−3.017626	−1.60523	−0.159852	C	−3.418175	−1.019408	−0.602379
O	3.986039	0.83685	−1.098164	O	3.909738	−0.891276	−1.651573
O	−1.622728	1.53867	0.692996	O	1.367802	3.232199	−0.394308
O	−2.875523	−0.999265	1.130251	O	−4.493899	−1.344099	0.292184
O	−0.178664	3.125258	0.02939	O	−0.375335	2.386689	0.762072
H	1.963379	2.371935	−0.626272	H	2.878038	1.45768	−1.231437
H	2.340049	−1.474169	−1.519803	H	1.375992	−2.20712	−1.327395
H	3.591079	−1.568817	−0.291386	H	2.715931	−2.728898	−0.317166
H	−0.391082	−0.662372	1.085889	H	−0.047656	0.07738	1.432652
H	0.571845	−3.208792	1.269019	H	−0.262068	−2.571372	2.009164
H	2.251219	−3.442904	0.767448	H	0.980314	−3.627287	1.323549
H	0.985784	−3.295322	−0.455766	H	−0.342716	−3.055069	0.302967
H	2.994501	−1.554759	2.157366	H	1.562298	−1.069436	2.847687
H	1.327681	−1.276535	2.68753	H	2.797116	−0.408251	1.766696
H	2.277359	0.056716	2.016067	H	2.77996	−2.139592	2.134582
H	−3.941617	−1.170689	−2.088312	H	−4.715972	−0.138256	−2.105515
H	−4.748623	−0.419904	−0.695027	H	−4.037965	1.031002	−0.952147
H	−3.227102	0.24971	−1.300982	H	−3.027698	0.358817	−2.253636
H	−1.47902	4.100833	1.34214	H	−1.063643	4.212271	0.01527
H	−2.069482	3.935855	−0.335068	H	−0.119215	4.313721	1.525621
H	−0.730427	5.072788	0.032486	H	−1.773821	3.619791	1.553005
H	−1.031929	−0.239373	−1.529708	H	−1.015747	0.388563	−1.115126
H	0.113817	−1.521267	−1.761943	H	−0.714108	−1.329813	−1.142883
H	−1.908481	−2.672948	−1.648873	H	−2.116395	−1.524499	1.009324
H	−1.304779	−2.885939	−0.015798	H	−2.447416	0.199288	0.881813
H	−3.644484	−2.488586	0.018948	H	−3.172847	−1.904265	−1.213684
H	−2.46743	−0.121641	0.997933	H	−5.267939	−1.558168	−0.253187
	**1A-5**				**1F-6**		
C	2.413211	0.131111	−0.81854	C	−1.737505	0.609014	0.975202
C	1.36357	0.668841	−0.166134	C	−0.853661	0.696013	−0.039744
C	2.616858	−1.326834	−0.91479	C	−2.292947	−0.684679	1.422635
C	1.288812	2.17121	−0.132659	C	−0.446094	2.041951	−0.562937
C	1.539478	−2.209867	−0.314895	C	−1.821016	−1.933791	0.700184
C	0.264205	−0.156271	0.476678	C	−0.285719	−0.518536	−0.748655
C	0.796146	−1.572726	0.882903	C	−1.324848	−1.691319	−0.745518
C	−0.341377	−2.514335	1.317969	C	−0.7117	−2.995477	−1.289107
C	1.764188	−1.429295	2.079346	C	−2.516835	−1.319403	−1.655391
C	−4.847982	0.155239	0.048153	C	4.228864	−1.233822	−0.414307
C	0.049892	4.042065	0.606631	C	−0.29521	4.357115	−0.121646
C	−0.979065	−0.157138	−0.472606	C	1.095822	−0.910673	−0.14614
C	−2.320748	0.000716	0.256364	C	2.128747	0.225526	−0.085542
C	−3.528853	−0.126372	−0.672328	C	3.506363	−0.193837	0.445378
O	3.604729	−1.7878	−1.479294	O	−3.098497	−0.732363	2.347688
O	2.113546	2.914422	−0.633924	O	−0.025787	2.224342	−1.692251
O	−3.508389	−1.464561	−1.195134	O	3.309591	−0.673624	1.784115
O	0.200906	2.610282	0.523334	O	−0.611251	3.02623	0.335872
H	3.159581	0.76636	−1.285619	H	−2.11941	1.493963	1.473666
H	0.82634	−2.429783	−1.123295	H	−1.009208	−2.359341	1.308407

H	1.993696	−3.166887	−0.035938	H	−2.634705	−2.667426	0.722511
H	−0.032706	0.356604	1.398759	H	−0.110243	−0.228252	−1.791418
H	−0.873264	−2.112262	2.187895	H	−0.283944	−2.842896	−2.28754
H	0.074499	−3.487114	1.606184	H	−1.487882	−3.765091	−1.373457
H	−1.071109	−2.689864	0.521272	H	0.073316	−3.390901	−0.637449
H	2.165841	−2.408535	2.364786	H	−3.268619	−2.117019	−1.64296
H	1.239132	−1.017192	2.948987	H	−2.182757	−1.189947	−2.691508
H	2.612005	−0.772234	1.860076	H	−3.008769	−0.392126	−1.345085
H	−4.875648	1.181677	0.431704	H	4.35568	−0.87247	−1.441471
H	−5.698473	0.029513	−0.63307	H	5.227493	−1.436354	−0.007965
H	−4.978626	−0.534905	0.889704	H	3.680086	−2.180723	−0.443138
H	−0.021277	4.47383	−0.394277	H	−0.941052	4.634631	−0.958027
H	0.899896	4.481394	1.133645	H	0.75023	4.41151	−0.43354
H	−0.872715	4.20445	1.162205	H	−0.475118	5.006999	0.733733
H	−0.889964	0.667818	−1.190043	H	0.959317	−1.310961	0.864618
H	−0.988565	−1.070939	−1.074871	H	1.498934	−1.723031	−0.757372
H	−2.423832	−0.746622	1.051833	H	2.264999	0.667966	−1.080008
H	−2.3506	0.985618	0.739564	H	1.764277	1.02668	0.56988
H	−3.414832	0.590686	−1.502384	H	4.129627	0.714759	0.47543
H	−4.235717	−1.538412	−1.833522	H	4.179731	−0.925167	2.132832
	**1A-7**				**1A-8**		
C	2.257318	−0.368808	−0.719028	C	1.698941	0.550992	−1.268521
C	1.349748	0.328627	−0.006787	C	0.800019	0.814675	−0.29848
C	2.098257	−1.812926	−0.985515	C	2.417246	−0.735375	−1.352601
C	1.546764	1.79136	0.267605	C	0.246462	2.212423	−0.268411
C	0.822949	−2.468398	−0.490333	C	2.097738	−1.781616	−0.299877
C	0.066137	−0.270557	0.532758	C	0.363677	−0.223672	0.720493
C	0.224535	−1.808227	0.774196	C	1.541905	−1.211829	1.026909
C	−1.121001	−2.48349	1.097038	C	1.084525	−2.380121	1.920452
C	1.165045	−2.042724	1.977975	C	2.656948	−0.453898	1.782362
C	−4.783467	1.386647	0.136764	C	−3.99864	−1.635297	0.560483
C	2.925834	3.684419	−0.03307	C	−0.804532	3.860734	1.057161
C	−1.111038	0.141595	−0.411376	C	−0.937565	−0.937726	0.247284
C	−2.390479	0.553754	0.330089	C	−2.110262	−0.005381	−0.093926
C	−3.560154	0.851458	−0.608421	C	−3.396281	−0.729635	−0.516248
O	2.958662	−2.434242	−1.60239	O	3.2342	−0.939354	−2.245912
O	0.762405	2.462948	0.916006	O	0.293121	2.991707	−1.203501
O	−3.87065	−0.378013	−1.284292	O	−3.085415	−1.472922	−1.704469
O	2.674749	2.284693	−0.268714	O	−0.28045	2.523148	0.928218
H	3.154404	0.098786	−1.110723	H	1.965256	1.308315	−1.999624
H	0.099832	−2.414851	−1.317817	H	1.363431	−2.464657	−0.751314
H	1.024498	−3.532341	−0.325032	H	3.001012	−2.377762	−0.127632
H	−0.120752	0.202936	1.503791	H	0.122769	0.298952	1.652686
H	−1.563967	−2.063573	2.007589	H	0.613018	−2.011894	2.839818
H	−0.965597	−3.555177	1.269609	H	1.949754	−2.987502	2.210924
H	−1.847504	−2.381245	0.284881	H	0.375117	−3.041088	1.413405
H	1.305518	−3.116425	2.149229	H	3.036872	0.404605	1.219657
H	0.735149	−1.612174	2.889837	H	3.502252	−1.121836	1.98457
H	2.153967	−1.596086	1.832794	H	2.284778	−0.083858	2.744968
H	−4.555687	2.335905	0.63525	H	−4.211962	−1.068663	1.47432
H	−5.614794	1.563942	−0.556572	H	−4.94389	−2.067138	0.209253
H	−5.116892	0.666971	0.893482	H	−3.325271	−2.461188	0.81149

H	3.011751	3.879466	1.038474	H	−1.613232	4.020597	0.340422
H	2.118332	4.290437	−0.450467	H	−0.013809	4.594487	0.885119
H	3.86677	3.898589	−0.538304	H	−1.176752	3.92858	2.078481
H	−0.798525	0.989729	−1.032871	H	−0.720502	−1.558617	−0.628967
H	−1.332225	−0.670547	−1.11094	H	−1.24806	−1.615166	1.047629
H	−2.706288	−0.229486	1.029414	H	−2.345631	0.637085	0.763429
H	−2.181971	1.450051	0.927853	H	−1.833323	0.659086	−0.922123
H	−3.237678	1.602697	−1.348725	H	−4.137849	0.048323	−0.760599
H	−4.572372	−0.187075	−1.927004	H	−3.896727	−1.926942	−1.983081
	**1A-9**				**1A-10**		
C	−2.128376	−0.133879	0.829514	C	−1.544355	0.715922	1.361794
C	−1.212195	0.39422	−0.006629	C	−0.664768	0.848847	0.34825
C	−2.12687	−1.567504	1.184383	C	−2.51202	−0.396795	1.429432
C	−1.254407	1.849828	−0.372585	C	0.172795	2.097648	0.352552
C	−0.996034	−2.413097	0.62992	C	−2.479486	−1.418624	0.306729
C	−0.068471	−0.398222	−0.609005	C	−0.516932	−0.190523	−0.749328
C	−0.439155	−1.91377	−0.724231	C	−1.891336	−0.893063	−1.024205
C	0.774373	−2.777243	−1.111155	C	−1.740304	−2.069689	−2.006512
C	−1.509855	−2.092737	−1.824593	C	−2.861724	0.126326	−1.662741
C	4.234212	−0.469172	1.102085	C	3.037796	−2.4317	1.22375
C	−2.339315	3.925705	−0.074724	C	1.468924	3.574345	−0.959013
C	1.233278	−0.074118	0.197914	C	0.631543	−1.181751	−0.395649
C	2.476044	0.121507	−0.684109	C	2.002059	−0.532032	−0.156079
C	3.741358	0.548964	0.070975	C	3.143555	−1.544499	−0.017239
O	−2.995798	−2.032907	1.915945	O	−3.301687	−0.483468	2.365239
O	−0.474591	2.367062	−1.154002	O	0.354312	2.801264	1.330111
O	3.454988	1.813165	0.688562	O	4.348345	−0.76449	0.019514
O	−2.239768	2.523238	0.243098	O	0.675277	2.373782	−0.862633
H	−2.9194	0.469834	1.26162	H	−1.60243	1.462912	2.147724
H	−0.198569	−2.406789	1.387958	H	−1.881698	−2.266518	0.671983
H	−1.344259	−3.449317	0.558571	H	−3.496247	−1.80264	0.166807
H	0.080966	−0.014644	−1.625105	H	−0.222467	0.326193	−1.66922
H	1.191487	−2.462392	−2.074571	H	−1.247564	−1.747676	−2.932105
H	0.468142	−3.825511	−1.209199	H	−2.728857	−2.460251	−2.274584
H	1.571159	−2.737194	−0.361846	H	−1.164677	−2.89835	−1.582914
H	−1.805531	−3.145496	−1.901694	H	−2.477198	0.467668	−2.631038
H	−1.112994	−1.784435	−2.798831	H	−3.016018	1.009702	−1.035179
H	−2.412918	−1.504747	−1.631586	H	−3.84034	−0.336156	−1.835561
H	4.435524	−1.438216	0.630652	H	3.003095	−1.817157	2.131042
H	5.167785	−0.124824	1.564068	H	2.142291	−3.061828	1.194855
H	3.498298	−0.615936	1.899492	H	3.905814	−3.098936	1.29441
H	−2.522817	4.061051	−1.143219	H	1.776929	3.634735	−2.001966
H	−1.419586	4.444272	0.205853	H	2.339651	3.504669	−0.303114
H	−3.181534	4.295442	0.50875	H	0.871363	4.445758	−0.682076
H	1.088526	0.849584	0.766387	H	0.342832	−1.76439	0.487417
H	1.402934	−0.857717	0.945166	H	0.730421	−1.891261	−1.224334
H	2.707907	−0.791722	−1.243422	H	2.249325	0.130378	−0.993967
H	2.25235	0.89858	−1.426054	H	1.989294	0.089991	0.749027
H	4.535623	0.686818	−0.680976	H	3.151389	−2.186381	−0.914228
H	4.250728	2.085989	1.172613	H	5.090637	−1.383635	0.108732
	**1A-11**				**1A-12**		
C	−1.475885	1.014345	1.035675	C	−1.479064	0.258073	1.202814

C	−0.641928	0.840012	−0.009791	C	−0.981086	0.587665	−0.006314
C	−2.422481	−0.034198	1.468652	C	−1.592588	−1.144476	1.65245
C	0.18066	1.989609	−0.512854	C	−0.948989	2.024187	−0.445398
C	−2.42425	−1.33904	0.691479	C	−1.04611	−2.216787	0.729764
C	−0.533262	−0.469071	−0.768119	C	−0.457771	−0.423007	−1.006037
C	−1.90608	−1.223634	−0.761848	C	−1.075578	−1.841579	−0.771469
C	−1.782467	−2.635066	−1.365041	C	−0.331929	−2.920744	−1.578553
C	−2.920822	−0.436066	−1.621007	C	−2.544958	−1.822692	−1.254695
C	3.169154	−2.069973	1.531142	C	4.254242	−0.698661	1.166789
C	1.11589	4.100455	−0.017633	C	−1.192273	4.283308	0.195796
C	0.651191	−1.315287	−0.215315	C	1.096235	−0.317294	−1.108158
C	2.015519	−0.610313	−0.234757	C	1.931665	−0.658108	0.134147
C	3.18838	−1.537544	0.097606	C	3.396596	−0.234419	−0.011082
O	−3.168075	0.154031	2.425377	O	−2.088991	−1.411122	2.743104
O	0.603225	2.063634	−1.653467	O	−0.746815	2.37071	−1.596544
O	4.373749	−0.76276	−0.138787	O	3.408465	1.197542	−0.116317
O	0.383695	2.936263	0.417663	O	−1.174703	2.887778	0.557675
H	−1.524229	1.955908	1.572961	H	−1.846229	1.012867	1.890335
H	−1.797315	−2.042064	1.259381	H	−0.015653	−2.412073	1.056001
H	−3.440101	−1.748977	0.71869	H	−1.605099	−3.141113	0.913724
H	−0.291531	−0.221318	−1.808464	H	−0.810521	−0.085834	−1.988965
H	−1.335845	−2.597106	−2.366109	H	−0.313739	−2.670456	−2.646088
H	−2.776446	−3.087168	−1.460992	H	−0.842083	−3.885342	−1.47237
H	−1.177167	−3.303943	−0.745673	H	0.701414	−3.056376	−1.24366
H	−2.582984	−0.384645	−2.662702	H	−2.593364	−1.624613	−2.331661
H	−3.065992	0.589373	−1.266768	H	−3.141123	−1.056119	−0.74801
H	−3.896823	−0.934807	−1.609726	H	−3.021592	−2.792485	−1.07021
H	3.165599	−1.239664	2.247188	H	3.864644	−0.292182	2.107385
H	2.289274	−2.695163	1.717734	H	4.266887	−1.792361	1.238339
H	4.056177	−2.685782	1.724836	H	5.29052	−0.358681	1.05039
H	1.183089	4.743886	0.858632	H	−1.377306	4.820696	1.124988
H	0.581297	4.6051	−0.82579	H	−1.988395	4.47844	−0.526533
H	2.112144	3.813634	−0.361857	H	−0.230861	4.575163	−0.232974
H	0.419565	−1.635978	0.807673	H	1.422472	−0.943535	−1.946818
H	0.72556	−2.224264	−0.822199	H	1.324734	0.713451	−1.396295
H	2.19931	−0.185928	−1.228847	H	1.524345	−0.161186	1.02478
H	2.033102	0.227608	0.475522	H	1.914998	−1.735902	0.332656
H	3.166038	−2.391997	−0.599654	H	3.799197	−0.671349	−0.940049
H	5.134817	−1.331428	0.060733	H	4.322754	1.466932	−0.298638

Table S3. Key transitions, oscillator strengths, and rotatory strengths in the ECD of conformers **1A**-1 at B3LYP /6-311++G(d,p) level.

Species	Excited State	ΔE (eV) [a]	λ (nm) [b]	f [c]	R_{vel} [d]
**1A**-1	69 -> 72	3.247	381.84	0.0001	−2.3494
	66 -> 70	4.3901	282.41	0.0049	−8.091
	63 -> 70	4.4733	277.17	0.0096	−7.0963
	63 -> 70	4.8057	257.99	0.2709	−2.2296
	63 -> 70	5.1063	242.81	0.012	−7.7866
	63 -> 70	5.4802	226.24	0.0475	38.273
	62 -> 70	5.5205	224.59	0.0186	−0.5332
	61 -> 70	5.592	221.72	0.0888	0.0218
	64 -> 70	5.8565	211.7	0.0277	0.2667
	69 -> 71	5.9834	207.21	0.0019	0.0081
	69 -> 72	6.1022	203.18	0.0028	0.0093
	59 -> 70	6.3628	194.86	0.0094	0.0043
	55 -> 70	6.3729	194.55	0.0198	−0.0219
	58 -> 70	6.5116	190.4	0.0095	0.0132
	59 -> 70	6.5137	190.34	0.0083	−0.3979
	67 -> 71	6.5522	189.22	0.0119	−0.0145
	56 -> 70	6.6723	185.82	0.023	−0.0409
	69 -> 72	6.7511	183.65	0.0031	0.4011
	69 -> 75	6.7754	182.99	0.0112	0.011
	67 -> 71	6.8395	181.28	0.0085	0.6752
	67 -> 72	6.8708	180.45	0.0084	0.0139
	66 -> 72	6.9316	178.87	0.007	0.0104
	66 -> 72	6.9467	178.48	0.0032	0.0494
	66 -> 72	6.9602	178.13	0.0026	−0.0198
	68 -> 72	7.0673	175.43	0.0077	−0.0961
	53 -> 70	7.1069	174.46	0.0093	0
	68 -> 74	7.1336	173.8	0.0089	−0.0524
	67 -> 72	7.1656	173.03	0.012	0.0199
	68 -> 73	7.1784	172.72	0.017	0.0126
	67 -> 72	7.2324	171.43	0.0032	0.036
	69 -> 77	7.2611	170.75	0.0006	0
	67 -> 72	7.2674	170.6	0.0264	0.3111
	68 -> 75	7.3044	169.74	0.0022	0.0208
	66 -> 71	7.3631	168.39	0.0225	0.0144
	52 -> 70	7.3773	168.06	0.0005	−0.0105
	67 -> 73	7.384	167.91	0.0046	0.1945
	69 -> 80	7.3919	167.73	0.0044	−0.0165
	69 -> 78	7.4093	167.34	0.0144	0
	67 -> 72	7.4722	165.93	0.0113	0.0252
	67 -> 75	7.4731	165.91	0.0045	−0.0169

[a] Excitation energy. [b] Wavelength. [c] Oscillator strength. [d] Rotatory strength in velocity form (10^{-40} cgs.).

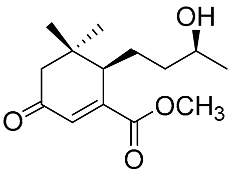

1B

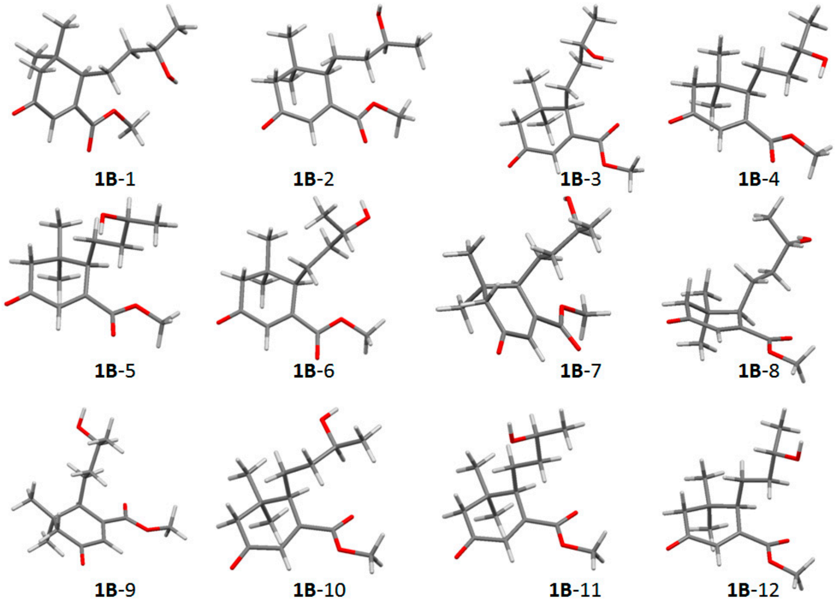

Figure S3. Optimized geometries of predominant conformers for **1B**at the B3LYP/6-31G(d,p) level in methanolsolution.

Table S4. Important thermodynamic parameters (a.u.) and Boltzmann distributions of the optimized **1B** at B3LYP/6-31G(d,p) level in methanol solution.

Conformations	E+ZPE	G	%
**1B−1**	−847.295001	−847.343457	5.49
**1B−2**	−847.293772	−847.341896	1.05
**1B−3**	−847.295502	−847.344551	17.47
**1B−4**	−847.293811	−847.342189	1.43
**1B−5**	−847.295696	−847.344319	13.67
**1B−6**	−847.293512	−847.342154	1.38
**1B−7**	−847.293493	−847.342106	1.31
**1B−8**	−847.293879	−847.343598	6.37
**1B−9**	−847.294524	−847.343463	5.52
**1B−10**	−847.294415	−847.343325	4.77
**1B−11**	−847.293772	−847.344860	1.05
**1B−12**	−847.294631	−847.344542	17.31

E+ZPE, G: total energy with zero point energy (ZPE) and Gibbs free energy in methanol solutionat B3LYP/6-31G(d,p) level. %: Boltzmann distributions, using the relative Gibbs free energies as weighting factors.

Table S5. Optimized Z-matrixes of **1B**in methanol solution (Å) at B3LYP/6-31G(d,p) level.

	**1B-1**				**1B-2**		
C	−2.292456	0.710887	−0.799405	C	−1.79865	1.330094	−0.736694
C	−1.149621	0.867913	−0.102416	C	−0.64836	0.935858	−0.152603
C	−2.90282	−0.61365	−1.021676	C	−2.957467	0.42908	−0.893946
C	−0.648774	2.276949	0.062859	C	0.383033	2.008016	0.061831
C	−2.136734	−1.815955	−0.504054	C	−2.816206	−0.988763	−0.368518
C	−0.349014	−0.288901	0.467503	C	−0.404406	−0.500346	0.27873
C	−1.27365	−1.52203	0.745626	C	−1.75131	−1.159978	0.740167
C	−2.191077	−1.206871	1.948965	C	−2.230832	−0.478029	2.041642
C	−0.463062	−2.782664	1.096097	C	−1.576321	−2.66012	1.043599
C	4.670146	−1.366133	0.073489	C	4.180125	−0.580348	−0.71198
C	1.045849	3.644631	0.978578	C	2.25777	2.676417	1.336044
C	0.872701	−0.557094	−0.473352	C	0.307799	−1.284218	−0.863864
C	2.18727	−0.840518	0.265171	C	1.709064	−0.796397	−1.270229
C	3.377327	−1.053694	−0.681696	C	2.813114	−1.075095	−0.236811
O	−3.968224	−0.722546	−1.621657	O	−3.98746	0.823391	−1.433026
O	−1.2084	3.264676	−0.378578	O	0.403476	3.073145	−0.52843
O	3.558799	0.065518	−1.564054	O	2.864957	−2.469333	0.105203
O	0.495939	2.328647	0.765534	O	1.267081	1.676286	1.019281
H	−2.814171	1.570416	−1.209084	H	−1.930381	2.356258	−1.066255
H	−2.851179	−2.623678	−0.311392	H	−3.802116	−1.324488	−0.027439
H	−1.495705	−2.159818	−1.32974	H	−2.562814	−1.619164	−1.233422
H	0.053363	0.037518	1.433189	H	0.26351	−0.490409	1.145846
H	−1.593761	−1.040809	2.853112	H	−2.380227	0.599809	1.923843
H	−2.867416	−2.047144	2.144594	H	−1.499372	−0.628155	2.844474
H	−2.804351	−0.314799	1.786394	H	−3.181895	−0.912761	2.37013
H	0.156712	−2.620065	1.985442	H	−0.754345	−2.82515	1.750707
H	0.188759	−3.101496	0.276707	H	−1.376466	−3.251062	0.144791
H	−1.145065	−3.612647	1.315476	H	−2.491831	−3.056191	1.498218
H	4.568381	−2.278997	0.671388	H	4.947961	−0.80421	0.035777
H	5.498793	−1.502648	−0.629006	H	4.464044	−1.069676	−1.653056
H	4.928746	−0.544884	0.754804	H	4.171168	0.501742	−0.886281
H	0.337863	4.26446	1.533425	H	2.867394	2.897179	0.457039
H	1.273676	4.119147	0.021344	H	2.866405	2.240196	2.126788
H	1.955552	3.488668	1.556822	H	1.771718	3.591021	1.682941
H	0.639298	−1.382711	−1.1576	H	0.409892	−2.327379	−0.554296
H	1.041598	0.313775	−1.113963	H	−0.333984	−1.274044	−1.753065
H	2.413404	0.005713	0.931255	H	1.986228	−1.310887	−2.202406
H	2.091836	−1.723169	0.907994	H	1.706487	0.273247	−1.515615
H	3.147023	−1.887855	−1.356716	H	2.565062	−0.578069	0.706437
H	3.775225	0.833435	−1.00855	H	3.105016	−2.952743	−0.703586
	**1B-3**				**1B-4**		
C	−2.257017	0.173229	−0.710036	C	−1.693549	0.876724	−1.202227
C	−1.188416	0.523697	0.033397	C	−0.747858	0.877654	−0.241092
C	−2.521583	−1.231199	−1.083349	C	−2.618197	−0.256541	−1.401198
C	−0.956307	1.955838	0.418853	C	0.035447	2.15114	−0.079356
C	−1.49123	−2.26196	−0.66202	C	−2.462649	−1.45293	−0.47883
C	−0.131925	−0.458379	0.500529	C	−0.478669	−0.324908	0.646461
C	−0.728721	−1.898298	0.633915	C	−1.801	−1.133892	0.882938
C	−1.699606	−1.936402	1.836229	C	−2.762105	−0.290279	1.75057
C	0.363768	−2.95341	0.882321	C	−1.53387	−2.45195	1.633348

C	4.996296	−0.204108	0.112229	C	3.619889	−2.375856	0.24172
C	−1.744185	4.178916	0.308336	C	1.381684	3.435071	1.376027
C	1.11596	−0.322812	−0.433757	C	0.680039	−1.187413	0.061012
C	2.461851	−0.346518	0.302218	C	1.995042	−0.438426	−0.202609
C	3.662055	−0.13637	−0.632401	C	3.121989	−1.330533	−0.750133
O	−3.523596	−1.530393	−1.726114	O	−3.469701	−0.222081	−2.284571
O	−0.003584	2.324222	1.084712	O	0.101885	3.029916	−0.92031
O	3.549292	1.092801	−1.367653	O	4.264802	−0.529234	−1.086334
O	−1.905218	2.789006	−0.038052	O	0.634031	2.228187	1.121165
H	−2.979971	0.907805	−1.048537	H	−1.839705	1.744831	−1.837826
H	−1.992521	−3.231558	−0.5693	H	−3.450392	−1.907514	−0.34272
H	−0.783478	−2.358393	−1.498925	H	−1.859939	−2.192752	−1.025867
H	0.1811	−0.135442	1.500357	H	−0.14158	0.044634	1.620904
H	−2.512758	−1.20968	1.741374	H	−2.320315	−0.097645	2.735299
H	−1.164677	−1.718971	2.768049	H	−3.705094	−0.826967	1.905854
H	−2.149471	−2.931381	1.933054	H	−2.999367	0.677015	1.296738
H	0.923318	−2.73413	1.799033	H	−0.961527	−3.166974	1.034517
H	1.075488	−3.019523	0.053462	H	−2.484616	−2.930783	1.894825
H	−0.09484	−3.942078	1.001961	H	−0.985677	−2.269693	2.565737
H	5.131793	−1.179709	0.59295	H	2.834961	−3.096719	0.489121
H	5.828428	−0.04052	−0.58035	H	4.464338	−2.925667	−0.186559
H	5.043424	0.565563	0.893865	H	3.955891	−1.895101	1.168188
H	−1.752297	4.302633	1.393803	H	0.722672	4.304885	1.32721
H	−2.594323	4.689712	−0.142013	H	2.184889	3.544127	0.643752
H	−0.805248	4.564476	−0.095748	H	1.788912	3.316938	2.379225
H	1.093493	−1.107542	−1.200802	H	0.873642	−1.998351	0.768724
H	1.063328	0.623095	−0.981288	H	0.34806	−1.656761	−0.874689
H	2.466432	0.447223	1.064079	H	1.825885	0.358843	−0.94047
H	2.597973	−1.29288	0.838487	H	2.35105	0.048565	0.71405
H	3.646864	−0.911837	−1.408968	H	2.756613	−1.844221	−1.655198
H	3.557152	1.8147	−0.716465	H	3.975999	0.127829	−1.740215
	**1B-5**				**1B-6**		
C	1.498769	0.722263	1.376581	C	−2.384933	0.329177	−0.838271
C	0.640517	0.855881	0.345015	C	−1.291988	0.750294	−0.171704
C	2.476608	−0.380917	1.453953	C	−2.742483	−1.098461	−0.941607
C	−0.212256	2.094191	0.343709	C	−1.05797	2.236079	−0.130516
C	2.477945	−1.391967	0.321135	C	−1.771375	−2.093236	−0.335191
C	0.528431	−0.172881	−0.766314	C	−0.29538	−0.188168	0.483698
C	1.916897	−0.856727	−1.017609	C	−0.97906	−1.54221	0.874225
C	2.891335	0.180109	−1.620649	C	−1.941292	−1.301139	2.059431
C	1.801842	−2.023224	−2.016535	C	0.047693	−2.599495	1.317794
C	−4.478794	−1.027719	0.162423	C	3.731218	−1.519606	−1.421872
C	−1.509609	3.56071	−0.977797	C	0.359067	3.960936	0.644185
C	−0.613669	−1.18247	−0.449773	C	0.96099	−0.306108	−0.444873
C	−2.001853	−0.570343	−0.215311	C	2.298964	−0.270057	0.309517
C	−3.084753	−1.637159	0.007659	C	3.537625	−0.244461	−0.599411
O	3.247547	−0.468206	2.405278	O	−3.768071	−1.449648	−1.517366
O	−0.416992	2.790561	1.322013	O	−1.78703	3.063926	−0.646982
O	−2.768656	−2.483924	1.123733	O	4.713461	0.039136	0.175234
O	−0.70293	2.369499	−0.876652	O	0.055388	2.554134	0.55161
H	1.531681	1.461704	2.171054	H	−3.05378	1.041848	−1.311044
H	3.500366	−1.767792	0.201717	H	−2.32712	−2.997853	−0.06536

H	1.876613	−2.246864	0.663529	H	−1.077268	−2.384206	−1.137888
H	0.250023	0.352175	−1.686596	H	0.036262	0.287111	1.413616
H	3.021141	1.057488	−0.97928	H	−2.446662	−2.233902	2.335313
H	2.526296	0.529017	−2.593802	H	−2.712462	−0.558339	1.831389
H	3.878791	−0.269901	−1.775514	H	−1.387579	−0.948642	2.937422
H	2.800531	−2.400376	−2.26569	H	−0.471009	−3.519826	1.611342
H	1.327366	−1.696115	−2.949894	H	0.622543	−2.25339	2.184489
H	1.225158	−2.862346	−1.615741	H	0.750749	−2.859516	0.520423
H	−5.221644	−1.814415	0.330119	H	2.894876	−1.691532	−2.108063
H	−4.509243	−0.338949	1.016867	H	4.649422	−1.449311	−2.014068
H	−4.765786	−0.464809	−0.73306	H	3.812706	−2.395216	−0.763845
H	−1.804643	3.622689	−2.024417	H	0.497111	4.384544	−0.353209
H	−0.926519	4.438014	−0.688825	H	1.282369	4.020835	1.218706
H	−2.388033	3.47768	−0.333738	H	−0.448891	4.488566	1.156125
H	−0.692317	−1.87705	−1.293335	H	0.879123	−1.213006	−1.054387
H	−0.347213	−1.782062	0.427917	H	0.959584	0.526867	−1.158313
H	−1.994212	0.093551	0.661778	H	2.327785	0.635066	0.928821
H	−2.291047	0.04954	−1.073457	H	2.381735	−1.125075	0.993883
H	−3.086846	−2.320577	−0.851083	H	3.454881	0.611336	−1.281683
H	−2.770665	−1.922532	1.917554	H	4.83919	−0.708011	0.783954
	**1B-7**				**1B-8**		
C	−1.56601	0.42496	1.366719	C	−2.265494	−0.248061	−0.731244
C	−1.205967	−0.357006	0.329111	C	−1.316441	0.37615	−0.00549
C	−1.283786	1.873106	1.407032	C	−2.207765	−1.697304	−1.011547
C	−1.610908	−1.805006	0.415535	C	−1.412361	1.846154	0.284476
C	−0.487624	2.456404	0.255627	C	−0.986641	−2.446847	−0.513532
C	−0.436714	0.150074	−0.875455	C	−0.082255	−0.31728	0.538397
C	−0.641433	1.690217	−1.080297	C	−0.350543	−1.842597	0.760545
C	−2.068718	1.922767	−1.629988	C	−1.313491	−2.018545	1.956887
C	0.354897	2.257357	−2.107085	C	0.939311	−2.61657	1.085917
C	4.137186	−0.285276	1.540603	C	4.077018	−0.22582	−1.524436
C	−1.785321	−3.852516	−0.751229	C	−2.644513	3.837738	−0.016666
C	1.032695	−0.380767	−0.839991	C	1.13032	0.033485	−0.388268
C	1.937964	0.062521	0.318266	C	2.431668	0.331411	0.372096
C	3.241799	−0.748171	0.397699	C	3.588136	0.815728	−0.516174
O	−1.664109	2.5573	2.352495	O	−3.104772	−2.249442	−1.64156
O	−2.086202	−2.328562	1.407086	O	−0.592174	2.451234	0.953569
O	4.017415	−0.619473	−0.804947	O	4.676391	1.277003	0.299879
O	−1.40632	−2.460747	−0.73951	O	−2.492115	2.425715	−0.264051
H	−2.110243	0.008722	2.20904	H	−3.124148	0.28604	−1.124074
H	−0.777173	3.506938	0.139475	H	−1.265679	−3.49477	−0.358568
H	0.56297	2.462441	0.575531	H	−0.256641	−2.440166	−1.336867
H	−0.874306	−0.334936	−1.756078	H	0.126294	0.130051	1.517038
H	−2.841378	1.52122	−0.965686	H	−0.855764	−1.635525	2.876367
H	−2.188179	1.443832	−2.608743	H	−1.538903	−3.079979	2.112344
H	−2.25975	2.994711	−1.755478	H	−2.263074	−1.493083	1.813422
H	0.284976	1.720717	−3.060902	H	1.419502	−2.22125	1.988267
H	1.391196	2.200246	−1.759037	H	1.665317	−2.583245	0.267357
H	0.130324	3.312366	−2.302821	H	0.702075	−3.670849	1.271398
H	3.619373	−0.377659	2.501015	H	4.927202	0.169191	−2.09006
H	5.051026	−0.887095	1.579105	H	4.401706	−1.140299	−1.009936
H	4.422252	0.764129	1.400999	H	3.291599	−0.50134	−2.236443

H	−2.853718	−3.954499	−0.547926	H	−2.735795	4.027613	1.05537
H	−1.216312	−4.40571	−0.000632	H	−3.557696	4.12544	−0.536063
H	−1.548794	−4.208459	−1.753012	H	−1.787106	4.386877	−0.41271
H	0.966524	−1.476539	−0.831969	H	1.278024	−0.771839	−1.116816
H	1.500991	−0.113307	−1.793433	H	0.881459	0.922426	−0.980623
H	2.208207	1.120837	0.216677	H	2.234004	1.117485	1.111416
H	1.41033	−0.046637	1.27463	H	2.765119	−0.553284	0.93057
H	2.9846	−1.80915	0.551627	H	3.262661	1.712304	−1.059231
H	3.542456	−1.082297	−1.512652	H	5.009504	0.50628	0.78939
	**1B-9**				**1B-10**		
C	1.604788	−0.389766	1.131872	C	−1.644566	1.391685	−0.516905
C	1.225626	0.15602	−0.041481	C	−0.548896	0.840312	0.044542
C	1.082638	−1.688766	1.603437	C	−2.839545	0.592669	−0.858138
C	1.841102	1.445067	−0.508121	C	0.562391	1.724884	0.526055
C	0.025745	−2.364409	0.751543	C	−2.794731	−0.895243	−0.557909
C	0.21042	−0.471951	−0.974351	C	−0.40759	−0.651464	0.281505
C	0.103189	−2.01863	−0.755149	C	−1.808912	−1.293985	0.565893
C	1.363144	−2.684446	−1.357	C	−2.332377	−0.787496	1.928985
C	−1.125644	−2.604579	−1.472737	C	−1.72685	−2.830041	0.643202
C	−3.775185	1.616936	1.611529	C	4.196326	−0.625664	−0.422173
C	3.197601	3.294565	0.053249	C	1.622976	3.826798	0.334837
C	−1.114191	0.354364	−0.966806	C	0.350812	−1.309587	−0.91043
C	−1.898449	0.454065	0.349294	C	1.782262	−0.811715	−1.180432
C	−3.055206	1.453512	0.272499	C	2.814407	−1.207326	−0.120717
O	1.482039	−2.174911	2.657535	O	−3.820567	1.124265	−1.36979
O	1.717348	1.87766	−1.641134	O	1.325271	1.41594	1.425589
O	−3.955672	0.96892	−0.73554	O	2.855149	−2.6444	−0.107205
O	2.55003	2.068211	0.447203	O	0.615437	2.90016	−0.120938
H	2.338049	0.091817	1.77013	H	−1.715719	2.45991	−0.694266
H	0.102976	−3.446212	0.908216	H	−3.813938	−1.225827	−0.328084
H	−0.94637	−2.064035	1.165175	H	−2.515919	−1.396327	−1.496439
H	0.603214	−0.328318	−1.988796	H	0.202707	−0.784018	1.181951
H	2.28876	−2.296248	−0.918402	H	−1.665071	−1.10655	2.738272
H	1.409294	−2.513952	−2.438751	H	−3.326724	−1.20156	2.132309
H	1.341747	−3.767333	−1.188519	H	−2.410038	0.303492	1.971409
H	−1.10704	−2.365506	−2.542842	H	−0.966852	−3.146433	1.367997
H	−2.067666	−2.23205	−1.058144	H	−1.48863	−3.286645	−0.322142
H	−1.129764	−3.696722	−1.376436	H	−2.690084	−3.238725	0.970351
H	−3.0957	2.003441	2.379902	H	4.544015	−0.948679	−1.410579
H	−4.610911	2.321472	1.518627	H	4.172137	0.470143	−0.401843
H	−4.175457	0.654838	1.951803	H	4.928935	−0.957608	0.323647
H	3.907161	3.106705	−0.755914	H	1.515257	4.705372	−0.299837
H	3.716018	3.646864	0.944085	H	2.617581	3.389446	0.221202
H	2.455465	4.026103	−0.274724	H	1.455022	4.085656	1.382753
H	−0.849274	1.369475	−1.287894	H	0.411089	−2.385127	−0.728346
H	−1.768547	−0.042583	−1.747697	H	−0.238393	−1.171376	−1.825407
H	−2.321675	−0.518489	0.629839	H	2.113524	−1.240579	−2.13609
H	−1.236139	0.762988	1.167552	H	1.807407	0.276557	−1.31493
H	−2.652514	2.432268	−0.038008	H	2.485893	−0.841939	0.862122
H	−4.665043	1.624546	−0.830381	H	3.421231	−2.909864	0.634805
	**1B−11**				**1B−12**		
C	1.430908	1.041514	1.041585	C	−1.647522	1.030595	−0.903122

C	0.617684	0.84952	−0.01702	C	−0.727263	0.809158	0.05745
C	2.396816	0.015286	1.484977	C	−2.543083	−0.037368	−1.393224
C	−0.229531	1.978288	−0.526394	C	0.044338	1.959617	0.633888
C	2.437345	−1.287994	0.70658	C	−2.385322	−1.417213	−0.779383
C	0.554179	−0.45759	−0.783426	C	−0.467524	−0.561591	0.652617
C	1.944626	−1.178839	−0.756182	C	−1.776088	−1.421718	0.643151
C	2.956469	−0.364662	−1.59369	C	−2.785086	−0.822593	1.648768
C	1.865218	−2.590569	−1.366383	C	−1.503573	−2.876495	1.069012
C	−4.502272	−0.973758	0.049207	C	3.728801	−2.335467	0.080773
C	−1.219852	4.065926	−0.038651	C	0.809558	4.170107	0.307115
C	−0.616306	−1.338201	−0.257234	C	0.735994	−1.244488	−0.061482
C	−1.998991	−0.669875	−0.261916	C	2.030161	−0.418828	−0.090783
C	−3.122408	−1.625997	0.143262	C	3.211862	−1.150056	−0.736116
O	3.126473	0.219909	2.450671	O	−3.372176	0.19496	−2.267983
O	−0.648269	2.040922	−1.669196	O	0.528912	1.949406	1.751915
O	−2.847162	−2.048079	1.487787	O	4.240124	−0.159484	−0.890346
O	−0.461575	2.920829	0.401896	O	0.128269	3.005714	−0.203948
H	1.446983	1.981896	1.582812	H	−1.804355	2.019075	−1.322668
H	3.459561	−1.67993	0.753506	H	−3.364589	−1.908654	−0.792525
H	1.810712	−2.002379	1.260427	H	−1.745878	−1.993826	−1.463783
H	0.326048	−0.208264	−1.82666	H	−0.17356	−0.408577	1.69786
H	2.63842	−0.320616	−2.641993	H	−2.381344	−0.863763	2.667182
H	3.944541	−0.838268	−1.563216	H	−3.719985	−1.394646	1.636056
H	3.068379	0.663839	−1.236086	H	−3.029633	0.221511	1.429065
H	1.436212	−2.558994	−2.375358	H	−0.996336	−2.912797	2.040892
H	1.263875	−3.275191	−0.760616	H	−0.889434	−3.417354	0.34253
H	2.871095	−3.019161	−1.44568	H	−2.450513	−3.419956	1.167573
H	−4.55273	−0.088569	0.693843	H	4.025761	−2.006638	1.083605
H	−4.724338	−0.668801	−0.979915	H	2.972415	−3.121399	0.181283
H	−5.284939	−1.673551	0.367016	H	4.603422	−2.785003	−0.405638
H	−1.309172	4.707692	0.836883	H	0.790186	4.895648	−0.505034
H	−2.206345	3.754379	−0.389552	H	1.838341	3.918739	0.574589
H	−0.6928	4.583906	−0.84336	H	0.288428	4.560643	1.184305
H	−0.670838	−2.233665	−0.886252	H	0.930486	−2.189969	0.452834
H	−0.397815	−1.679473	0.760533	H	0.455194	−1.497105	−1.09269
H	−2.020546	0.182687	0.430622	H	1.877815	0.50331	−0.665435
H	−2.222776	−0.275631	−1.260554	H	2.314237	−0.116386	0.925482
H	−3.096214	−2.502059	−0.526385	H	2.900706	−1.510565	−1.730993
H	−3.526364	−2.69679	1.732425	H	5.006077	−0.599008	−1.293581

Table S6. Key transitions, oscillator strengths, and rotatory strengths in the ECD of conformers **1B**-3 at B3LYP /6-311++G(d,p) level.

Species	Excited State	ΔE (eV) [a]	Λ (nm) [b]	f [c]	R_{vel} [d]
**1B**-3	68 -> 70	3.2168	385.43	0.0001	−4.2077
	62 -> 70	4.3536	284.79	0.0549	17.0939
	68 -> 70	4.3759	283.33	0.0097	6.1204
	64 -> 70	4.7729	259.77	0.2234	0.0729
	64 -> 70	5.024	246.78	0.0016	20.8716
	62 -> 70	5.3786	230.51	0.0725	−8.4712
	64 -> 70	5.5124	224.92	0.0212	8.8054
	64 -> 70	5.5825	222.09	0.0811	−0.9651
	67 -> 70	5.6706	218.65	0.0348	0.2197
	69 -> 71	5.948	208.45	0.0027	0.0122
	69 -> 72	6.0393	205.29	0.0066	0.0113
	56 -> 70	6.1768	200.73	0.0061	0.009
	69 -> 80	6.3865	194.14	0.017	−0.0149
	57 -> 70	6.4191	193.15	0.03	−0.016
	55 -> 70	6.437	192.61	0.0123	−0.0112
	68 -> 73	6.5648	188.86	0.0208	0.0119
	69 -> 71	6.7163	184.6	0.0121	−0.0347
	57 -> 70	6.7317	184.18	0.0117	−0.0162
	69 -> 71	6.8013	182.3	0.0002	0.0373
	68 -> 73	6.853	180.92	0.0141	−0.1055
	68 -> 72	6.9022	179.63	0.0055	0.0124
	69 -> 74	6.9242	179.06	0.0021	−0.0164
	55 -> 70	6.9435	178.56	0.0086	−0.0394
	68 -> 72	7.0087	176.9	0.0035	0.0126
	68 -> 73	7.0419	176.07	0.0085	−0.0622
	69 -> 75	7.0613	175.58	0.0115	−0.1077
	67 -> 72	7.1327	173.83	0.0005	−0.0165
	54 -> 70	7.1763	172.77	0.0161	0.0216
	67 -> 72	7.1863	172.53	0.0095	−0.3536
	68 -> 75	7.1984	172.24	0.0035	−0.0104
	69 -> 77	7.2455	171.12	0.0035	0.0283
	69 -> 76	7.2561	170.87	0.0167	0.0357
	68 -> 76	7.323	169.31	0.0084	0.031
	69 -> 81	7.3331	169.07	0.0171	−0.0116
	68 -> 77	7.3683	168.27	0.0014	0.1265
	66 -> 74	7.3814	167.97	0.0029	−0.0444
	69 -> 81	7.4288	166.9	0.009	−0.0106
	69 -> 82	7.479	165.78	0.0047	0.0377
	50 -> 70	7.5205	164.86	0.0073	−0.2997
	53 -> 70	7.5237	164.79	0.002	−0.0104

[a] Excitation energy. [b] Wavelength. [c] Oscillator strength. [d] Rotatory strength in velocity form (10^{-40} cgs.).

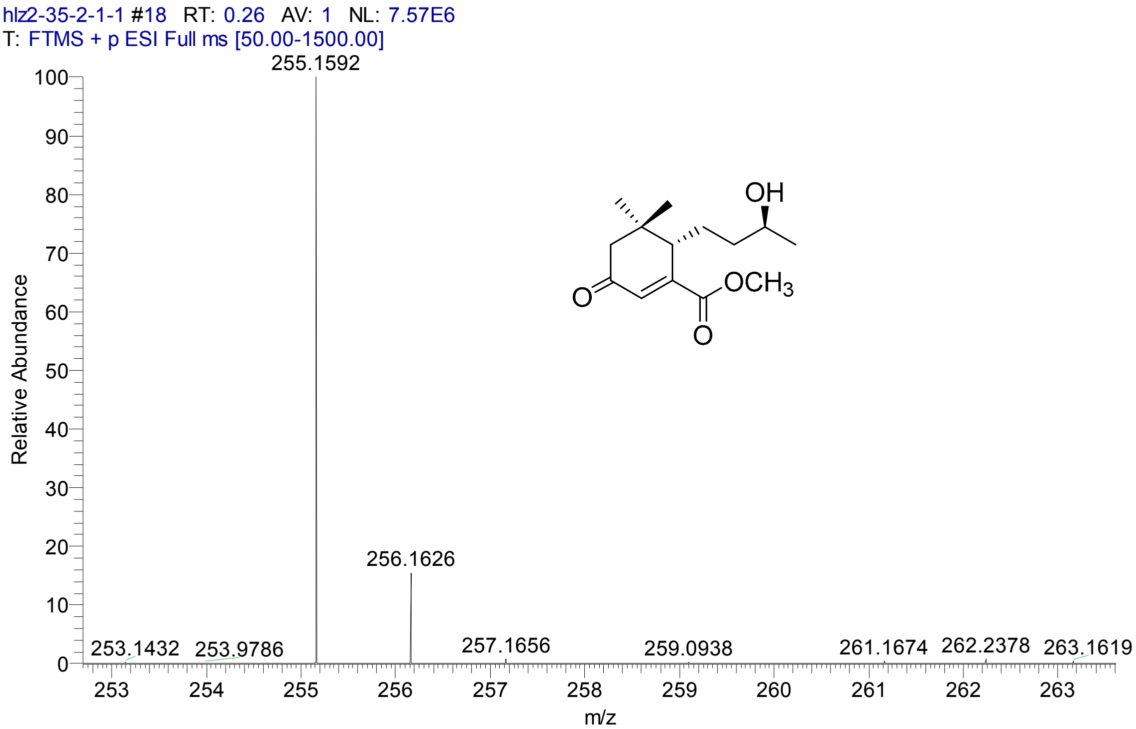

Figure S4. HRESIMS spectrum of schinifolenol A(**1**).

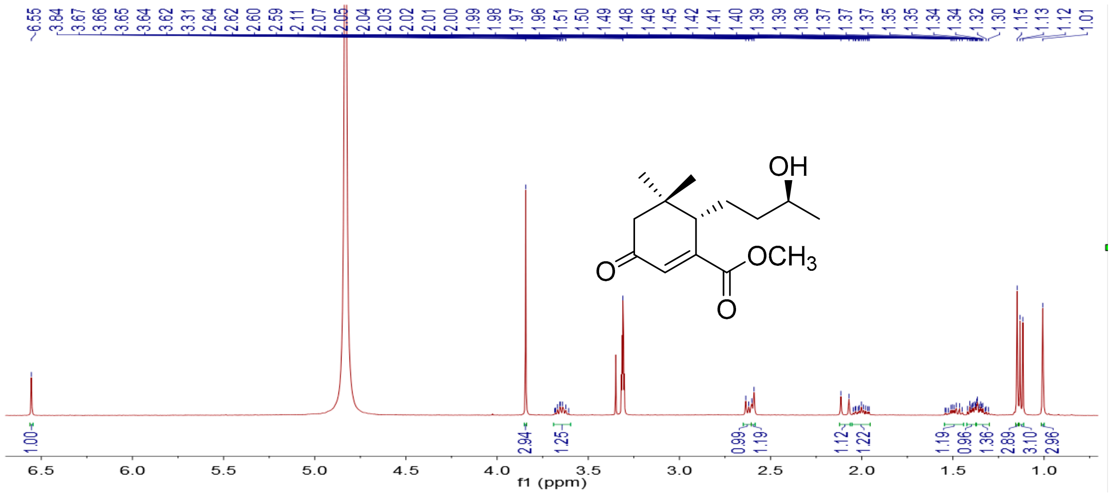

Figure S5. ^{1}H-NMR spectrum of schinifolenol A(**1**, in methanol-d_4).

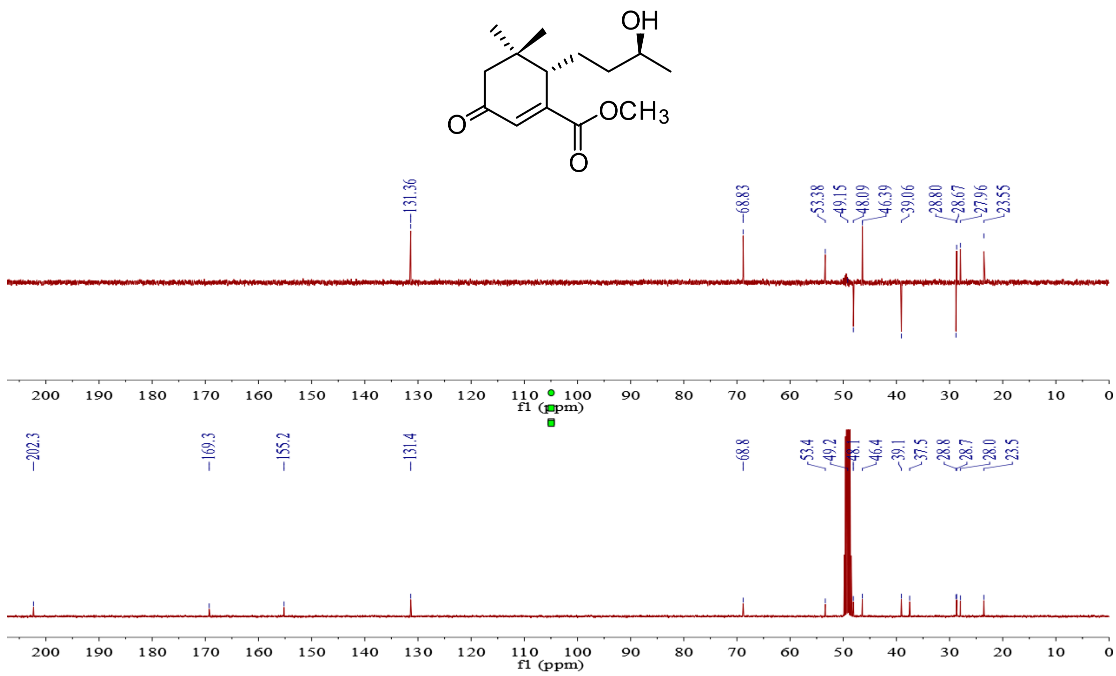

Figure S6. ^{13}C-NMR and DEPT135 spectra of schinifolenol A(**1**, in methanol-*d*$_4$)

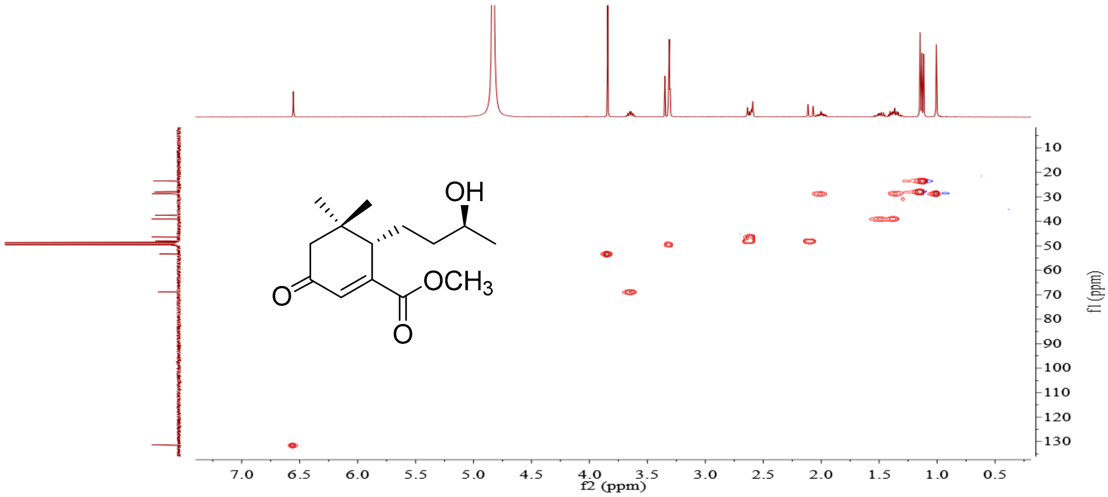

Figure S7. HSQC spectrum of schinifolenol A(**1**, in methanol-*d*$_4$).

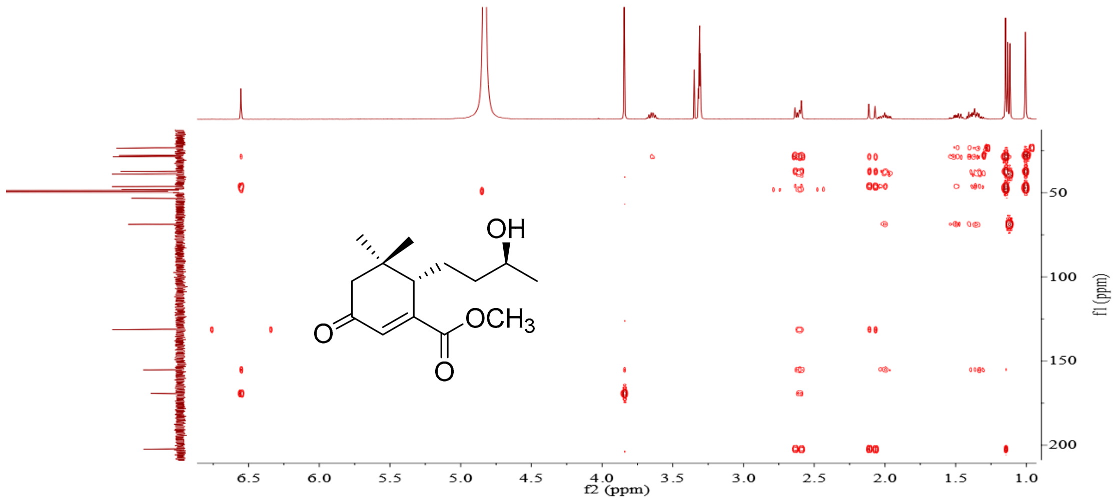

Figure S8. HMBC spectrum of schinifolenol A(**1**, in methanol-*d*$_4$).

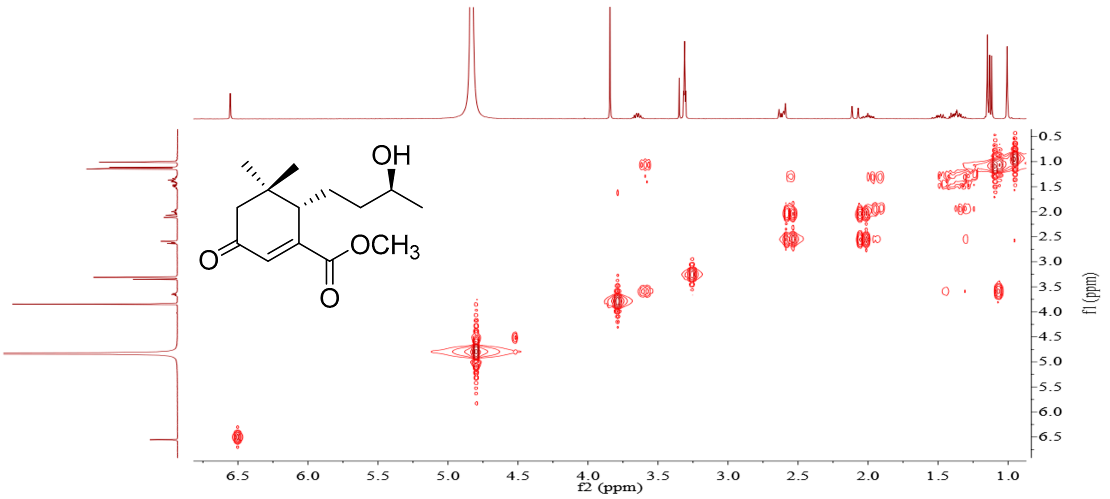

Figure S9. ^{1}H–^{1}H COSY spectrum of schinifolenol A(**1**, in methanol-*d*4).

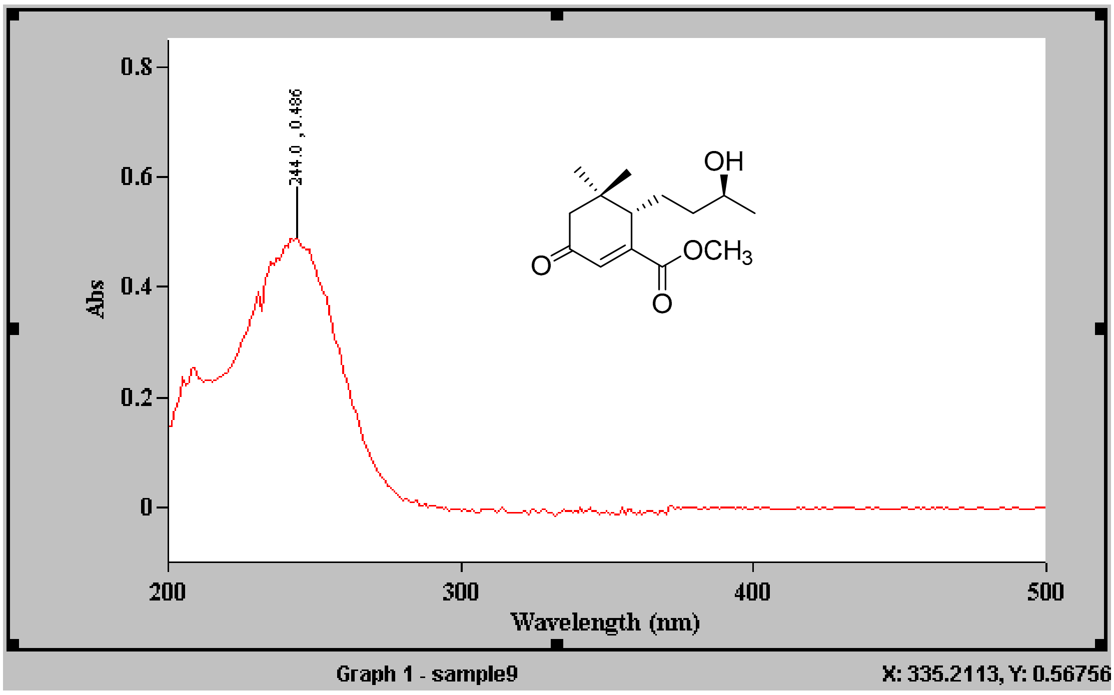

Figure S10. UV spectrum of schinifolenol A (1, in methanol-*d*4).

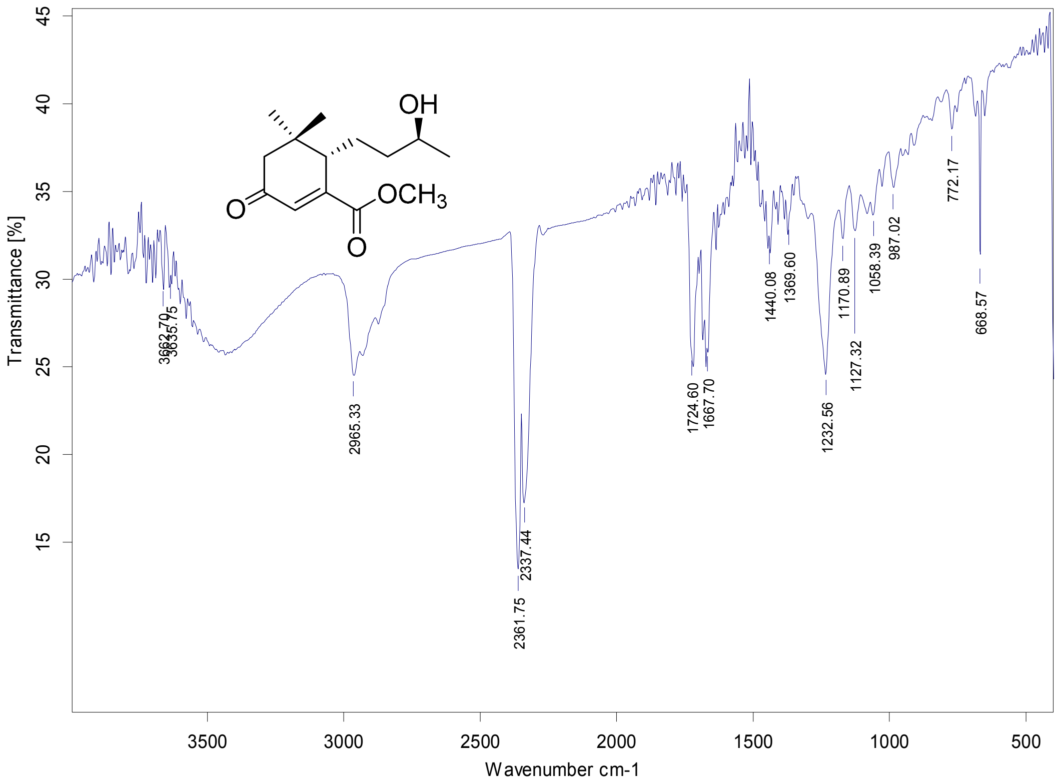

Figure S11. IR spectrum of schinifolenol A (**1**, in methanol-d_4).

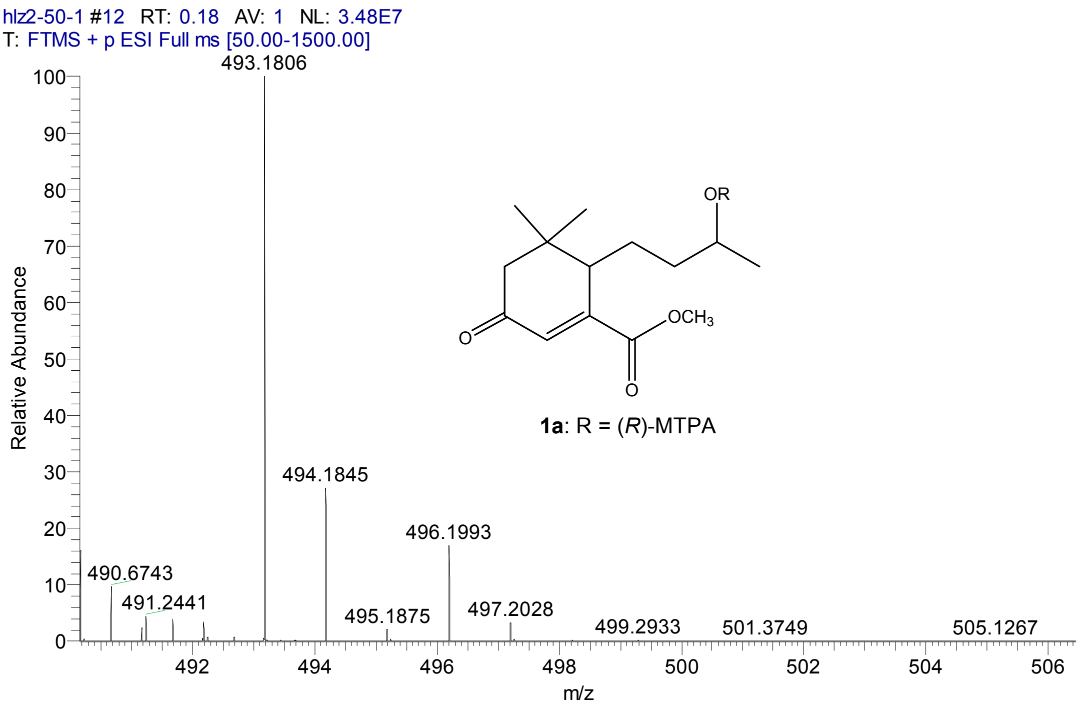

Figure S12. HRESIMS spectrum of compound **1a**.

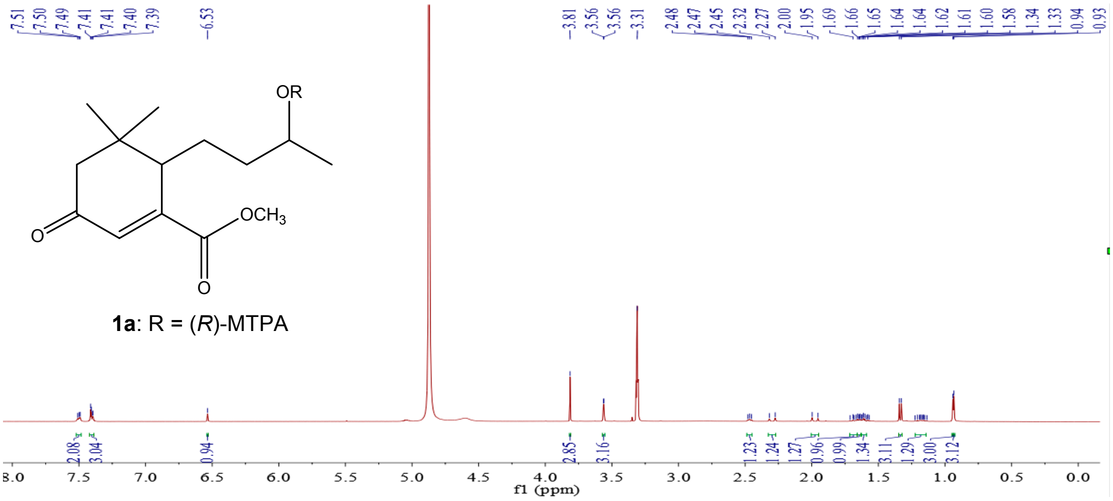

Figure S13. ^{1}H-NMR spectrum of compound**1a** (in methanol-d_4).

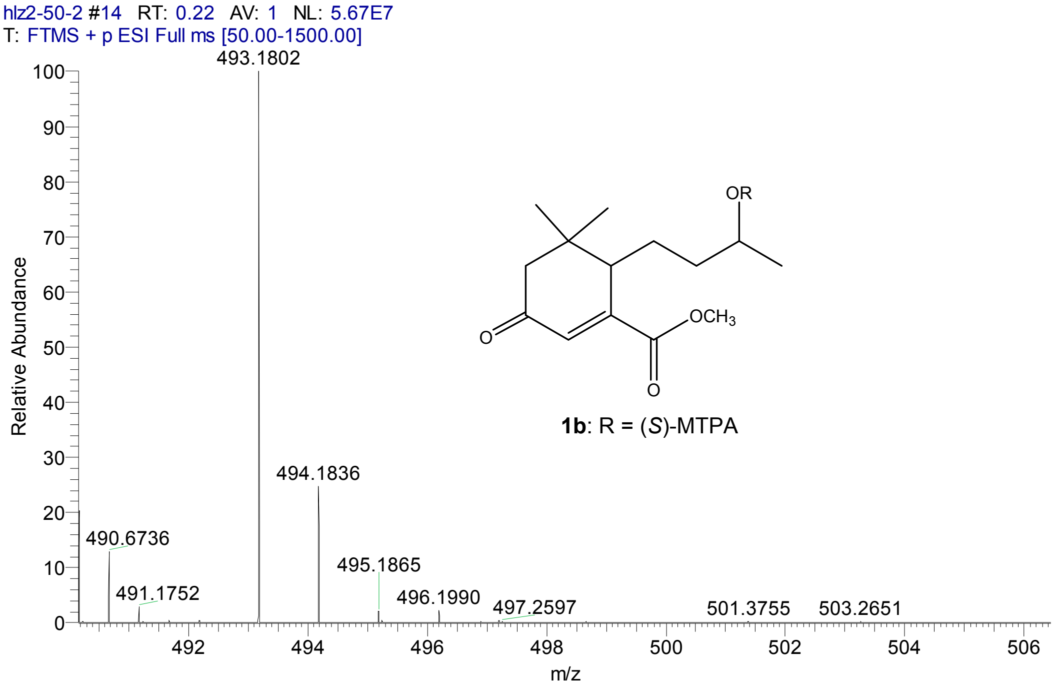

Figure S14. HRESIMS spectrum of compound **1b**.

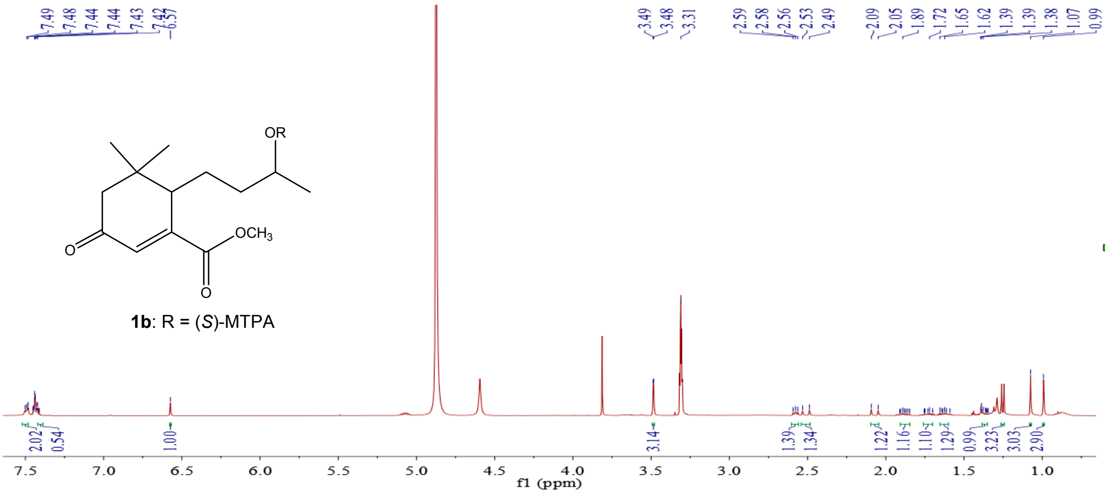

Figure S15. ^{1}H-NMR spectrum of compound**1b** (in methanol-d_4).

References

1. Vainio, M.J.; Johnson,M.S. Generating conformer ensembles using a multiobjective genetic algorithm. *J. Chem. Inf. Model.* **2007**, *47*, 2462–2474.
2. O'Boyle, N.M.; Vandermeersch, T.; Flynn, C.J.;Maguire, A.R.; Hutchison, G.R. Confab-systematic generation of diverse low-energy conformers. *J. Cheminform.* **2011**, *3*, 1–9.
3. *Gaussian 09, Revision C.01*; Frisch, M.J.; Trucks, G.W.; Schlegel, H.B.; Scuseria, G.E.; Robb, M.A.; Cheeseman, J.R.; Scalmani, G.; Barone, V.; Mennucci, B.; Petersson, G.A.; *et al.* Gaussian, Inc.: Wallingford CT, USA, 2013.
4. Tomasi, J.; Mennucci, B.; Cammi, R. Quantum mechanical continuum solvation models. *Chem. Rev.* **2005**, *105*, 2999–3094.
